# Activity-dependent diffusion trapping of AMPA receptors as a key step for expression of early LTP

**DOI:** 10.1098/rstb.2023.0220

**Published:** 2024-07-29

**Authors:** Agata Nowacka, Angela M. Getz, Diogo Bessa-Neto, Daniel Choquet

**Affiliations:** ^1^ University of Bordeaux, CNRS, Interdisciplinary Institute for Neuroscience, IINS, UMR 5297, Bordeaux F-33000, France; ^2^ University of Bordeaux, CNRS, INSERM, Bordeaux Imaging Center, BIC, UMS 3420, US 4, Bordeaux F-33000, France

**Keywords:** long-term potentiation, synaptic plasticity, AMPAR trafficking, AMPAR diffusion trapping

## Abstract

This review focuses on the activity-dependent diffusion trapping of α-amino-3-hydroxy-5-methyl-4-isoxazolepropionic acid receptors (AMPARs) as a crucial mechanism for the expression of early long-term potentiation (LTP), a process central to learning and memory. Despite decades of research, the precise mechanisms by which LTP induction leads to an increase in AMPAR responses at synapses have been elusive. We review the different hypotheses that have been put forward to explain the increased AMPAR responsiveness during LTP. We discuss the dynamic nature of AMPAR complexes, including their constant turnover and activity-dependent modifications that affect their synaptic accumulation. We highlight a hypothesis suggesting that AMPARs are diffusively trapped at synapses through activity-dependent interactions with protein-based binding slots in the post-synaptic density (PSD), offering a potential explanation for the increased synaptic strength during LTP. Furthermore, we outline the challenges still to be addressed before we fully understand the functional roles and molecular mechanisms of AMPAR dynamic nanoscale organization in LTP.

This article is part of a discussion meeting issue ‘Long-term potentiation: 50 years on’.

## 1. Introduction

Activity-dependent changes in the efficacy of synaptic transmission, and first and foremost, long-term potentiation (LTP), have been a central focus in neuroscience since their initial description by Bliss and Lomo [1]. Since its discovery, LTP has been suggested to represent a potential cellular substrate of learning and memory, promoting the extensive investigation of the sequence of biophysical and biochemical events underlying its induction and expression. Two broad categories of LTP have been described in terms of expression. Those that are expressed pre-synaptically as a sustained change in glutamate release [1–5], and those that are expressed post-synaptically [6–8]. Both pre- and post-synaptic forms of LTP are, however, for the vast majority, induced post-synaptically through activation of *N*-methyl-d-aspartate receptors (NMDARs) [9,10]. The post-synaptic forms of synaptic potentiation represent the majority of LTP types and have been shown to be essentially mediated by increases in synaptic responsiveness mediated by α-amino-3-hydroxy-5-methyl-4-isoxazolepropionic acid receptors (AMPARs), the major excitatory neurotransmitter receptors in the brain. However, 50 years after its discovery and despite decades of intense work, it is still unclear how LTP induction triggers this increase in AMPAR responses. It has been suggested that this may result from a change in AMPAR biophysical properties [11–13] and/or an increased AMPAR accumulation at synapses [14,15]. In any case, we still do not know precisely how LTP induction leads to the stable trapping of AMPARs at the synapse to establish and maintain increased synaptic strength.

An important turning point in our understanding of the cellular events responsible for increased AMPAR responsiveness during LTP occurred at the turn of the century when a series of papers established that neurotransmitter receptors are not stable in the post-synaptic membrane but undergo constant turnover through endocytic and exocytic processes [16–23]. Concomitantly, many studies at that time established that activity-dependent modifications in AMPAR trafficking lead to changes in their accumulation in front of the transmitter release site (reviewed in [24–28]) all the way from cell culture systems (e.g. [29,30]) up to *ex vivo* brain slices (e.g. [31,32]) and even *in vivo* [33–35].

An initial focus was put on the role of AMPAR exocytosis in supplying new receptors for LTP, prompted by the observation of activity-dependent AMPAR exocytosis [36–39] and studies showing that blocking exocytosis could inhibit LTP. Further interest was raised for exocytosis mechanisms because they potentially allow changes to the subtype of receptors on the cell surface [40–43]. However, two main lines of evidence suggested that AMPAR exocytosis might not be the principal mechanism for increased AMPAR responsiveness during LTP. First, AMPAR exocytosis was primarily observed in dendritic shafts [36–39,44]. Second, subsequent evidence, mostly from our team, but also others, indicated the presence of a significant population of extrasynaptic AMPARs capable of being reversibly trapped at synapses in an activity-dependent manner [30,31,45–49]. Altogether, this led us to propose a leading hypothesis for LTP a decade ago, according to which plasma membrane-inserted AMPARs are diffusively trapped at synapses through activity-dependent increased binding to protein-based binding ‘slots’ in the PSD [27,50].

However, the exact molecular players and mechanisms responsible for the increased AMPAR responsiveness, as well as the respective roles of the various AMPAR trafficking pathways and, first and foremost, AMPAR diffusion trapping and exocytosis, are not yet fully uncovered. This is largely due to the difficulty of combining *bona fide* LTP experiments and imaging of receptor trafficking. The development of new tools to manipulate, and image, AMPAR trafficking and nanoscale dynamics is, however, providing new insights into the fascinating molecular mechanism of LTP. We will review here the elements that support the key role of activity-dependent AMPAR diffusion trapping in LTP expression, particularly at early stages, the underlying potential molecular mechanisms and the many remaining unknowns.

## 2. Mechanisms of increased AMPAR responsiveness during LTP

If there is one certainty in the field of LTP, it is that most, if not all, of its post-synaptic forms are associated with increased post-synaptic AMPAR responsiveness. However, what actually mediates this increased responsiveness is still not completely clear. Two broad categories of hypotheses have been proposed: alterations in the biophysical characteristics of AMPARs, a change in AMPAR numbers, or a combination of both factors. Prior to demonstrating the highly dynamic nature of AMPAR exchange between synaptic and extrasynaptic sites, the prevailing view was that AMPARs, like all neurotransmitter receptors, remained at synaptic sites for extended periods of time, roughly equivalent to the protein’s lifespan of several days. As a consequence, once the exploitation of the silent synapse potentiation paradigm had indeed established that LTP at the canonical Schaffer collateral to the CA1 pyramidal neuron synapse was associated with an increased AMPAR responsiveness [7,8], one of the first mechanisms that was proposed to account for this increase was the change in AMPAR biophysical properties, without actual changes in receptor numbers or types [11–13].

Indeed, it was established that phosphorylation of the AMPA receptor subunit GluA1 by Ca2+/calmodulin-dependent protein kinase II (CaMKII) plays a crucial role in regulating synaptic strength through an increase in the proportion of large conductance single-channel openings [12,21,51–57]. Mutations mimicking phosphorylated (S831D/E) and unphosphorylated (S831A) states of GluA1 display corresponding changes in channel conductance. Non-CaMKII sites on GluA1’s C-tail, phosphorylated by protein kinase C (PKC), also impact receptor function, because dephosphorylation reduces AMPAR currents [58]. In addition, protein kinase A (PKA) phosphorylation of GluA1 at Ser845 increases receptor peak open probability without altering single-channel conductance, suggesting a two-state model of receptor functionality [57,59]. More recent work indicated that the phosphorylation state of GluA1 influences the coupling efficiency of GluA1/2 heteromers, especially in the presence of AMPAR auxiliary subunits such as transmembrane AMPA receptor regulatory proteins (TARPs). Structural insights suggest that phosphorylation at Ser831 modifies local secondary structure, potentially influencing interactions with other proteins or the plasma membrane and, thus, channel properties. However, the AMPAR C-terminus is largely unstructured, and it is thus still unclear how changes in its phosphorylation could affect the channel properties (reviewed in [60]). An alternative hypothesis could be that post-translational modifications in GluA1 c-terminus could impact AMPAR intracellular transport, as shown recently [61], thereby modifying the AMPAR subtype present in synapses.

The second and most actively investigated substrate for enhanced AMPAR responsiveness during LTP is an increase in AMPAR numbers. This has been established in a variety of ways and preparations [24,62,63]. For example, in dendrites visualized with two-photon laser scanning microscopy, tetanic synaptic stimulation induced a rapid, *N*-methyl-d-aspartate (NMDAR)-dependent, delivery of green fluorescent protein (GFP)-tagged GluA1 into dendritic spines as well as into clusters in dendrites [32]. Similarly, LTP-induced delivery of GluA1 AMPARs into synapses was shown using an electrophysiological tag of receptors [64]. Repetitive quantum-like photorelease (uncaging) of glutamate-simulating LTP induction has consistently demonstrated the induction of rapid and specific enlargement of stimulated spines and is associated with an increase in α-amino-3-hydroxy-5-methyl-4-isoxazolepropionic acid (AMPA)R-mediated currents at the stimulated synapse, contingent upon NMDARs' activation [65].

Subunit-specific rules governing AMPA receptor trafficking to synapses in hippocampal pyramidal neurons have been identified early [66]. Using cleavable extracellular tags, it has been established that surface insertion of AMPARs along dendrites occurs in a subunit-dependent manner [44]. Using overexpression approaches, it was suggested that new GluA1-containing AMPARs are diffusively distributed along dendrites. This would be followed by their lateral translocation and accumulation into synapses. By contrast, GluA2 subunits would accumulate directly at synapses, pointing to local exocytosis taking place. It should be noted, however, that direct exocytosis in the PSD has never been firmly established (however, see [57,67]). Rather, most studies have reported a strong bias towards dendritic exocytosis [36–38,44] with occasional direct spine exocytosis [39,68]. All of these studies have relied on the overexpression of tagged AMPAR subunits, without carefully controlling the expression levels. Considering our current understanding that AMPARs are made up of a core pore-forming tetramer consisting of subunits GluA1–GluA4, which is then surrounded by several auxiliary subunits (from almost a dozen that have been identified) with diverse regulatory functions, it is highly probable that the behaviour of naturally occurring receptors differs significantly from these overexpressed, and thus simplified, versions of the receptors.

In a broader context, while early investigations primarily focused on exocytosis as the main source for AMPAR accumulation at synapses during LTP, evidence indicating the diffusive nature of AMPARs on the neuronal surface, along with their reversible trapping at synapses in an activity-dependent manner [30,31,45–49] (reviewed in [28]), pointed to an important potential role of diffusion trapping in regulating AMPAR accumulation at synapses upon LTP induction. Using an approach to acutely manipulate AMPAR surface diffusion through cross-linking them using either antibodies or tetravalent avidin for biotinylated receptors, we could establish that AMPAR surface diffusion is mandatory for the expression of early phases of LTP [69,70]. It would seem that AMPAR exocytosis is not required for this early LTP expression (seconds to minutes after the induction protocol) but rather it is necessary for LTP maintenance. This is consistent with the fact that AMPAR exocytosis sites are remote from the PSD [30,71,72], and newly exocytosed receptors are expected to take minutes to diffuse to the PSD. However, one should note that there is an unresolved conundrum in the fact that, at rest, a sizeable pool of extrasynaptic mobile receptors is already present and that only a few exocytotic vesicles, containing probably a very limited number of AMPARs, are observed upon strong LTP-like stimuli. Therefore, it is quite unlikely that mild physiological LTP stimuli would trigger a sufficiently large AMPAR exocytosis meaningful enough to substantially alter extrasynaptic AMPAR density. That said, while an intact post-synaptic exocytotic machinery has been robustly demonstrated to be required for LTP expression [40–43], AMPAR exocytosis has not yet been observed during physiologically triggered LTP. Interestingly, it has been proposed that the requirement for an intact exocytic machinery does not imply a requirement for AMPAR exocytosis, but that, alternatively, LTP maintenance may require exocytosis of a yet-to-be-identified factor such as EphB [73].

An important question is whether increased AMPAR responsiveness during LTP is due to a mere increase in the number of pre-existing AMPAR types, or if exchange in AMPAR subtypes is involved. At canonical synapses such as the Shaffer collateral to the CA1 pyramidal neuron synapse, the main subtype of AMPARs present are calcium-impermeable (CI-AMPARs) GluA1/A2 heteromers [74]. While there is a wide consensus that calcium-permeable GluA2-lacking receptors (CP-AMPARs) do not participate in basal synaptic transmission in these neurons, there have been conflicting results as to their implication in LTP. Various lines of evidence suggest that GluA1 homomers can be inserted transiently at synapses by LTP stimuli, with their Ca^2+^ signal contributing to the expression of LTP [57,59]. The application of CP-AMPAR inhibitors shortly, but not later than 30 min, after the delivery of an LTP-inducing stimuli largely reverses LTP [57,75–77]. This led to hypothesis that there is a short time window after LTP triggering when the incorporation of CP-AMPARs is required for LTP expression [78]. However, other groups found no such effect [79,80], resulting in an uncertainty on the role of CP-AMPARs in LTP.

A safe statement at this point would be that the involvement of CP-AMPARs in LTP strongly depends on the type of LTP-inducing stimuli used and the preparation type [57], weaker stimuli being most likely to involve the transient recruitment of CP-AMPARs. The canonical CP-AMPARs are GluA1 homomers that exhibit a higher single-channel conductance than GluA1/A2 heteromers. Hence, transient CP-AMPARs' incorporation in synapses upon LTP induction would automatically increase synaptic currents. A plausible scenario could thus be that initial synaptic potentiation upon LTP induction by certain stimuli is owing to a switch from CI-AMPARs to CP-AMPARs. This would then be followed by the addition of regular CI-AMPARs for sustained LTP [78]. This concept was recently elegantly established by Park *et al*. [59], who demonstrated that LTP induced by compressed theta burst stimulation (TBS), with a 10 s inter-episode interval, involves purely an increase in the number of AMPARs. In contrast, either a spaced TBS, with a 10 min inter-episode interval, or a single TBS, delivered when PKA is activated, results in LTP that is associated with a transient increase in AMPAR single-channel conductance, caused by the insertion of CP-AMPARs. It should be noted that we could block LTP induced both by high-frequency stimulation and TBS by cross-linking surface GluA2 [69]. It will be interesting to investigate this issue further using various TBS protocols and cross-linking either GluA1- or GluA2-containing AMPARs. Importantly, along this line, it was shown that LTP requires a reserve pool of glutamate receptors independent of subunit type [81]. Resolving the question of the involvement of CP-AMPARs in LTP shall await further experiments and the capability to acutely label and manipulate CP- and CI-AMPARs differentially.

In any case, while evaluating the increased AMPAR responsiveness during LTP has often been reduced to visualizing/measuring AMPAR numbers at synapses, the topic emerges as being more complex. Recent studies using optogenetics or modelling to artificially recruit AMPARs at the PSD [82,83] established that, surprisingly, adding more AMPARs to excitatory contacts had little effect on synaptic strength. Instead, the authors observed increased excitatory input through the apparent addition of new functional sites. This evidence indicates that introducing more AMPARs can effectively activate synapses with a sparse initial number of receptors. Yet, for the reinforcement of established synapses, additional remodelling events are required to strengthen them through precise receptor positioning within PSD subdomains. A clue could come from the existence of small nanoscale re-organizations of receptors within the PSD allowing activity-dependent changes in AMPAR alignment to release sites without the need for a change in net receptor numbers. Long-term synaptic plasticity would thus require a redistribution of AMPARs through lateral diffusion to modulate their number and nanoscale organization. Indeed, in the past 10 years, there has been a major evolution in our understanding of synapse nanoscale organization and its functional consequences thanks to the advent of superresolution microscopy, as reviewed below.

## 3. Post-synaptic nano-organization and trans-synaptic complexes alignment

**Figure 1.**
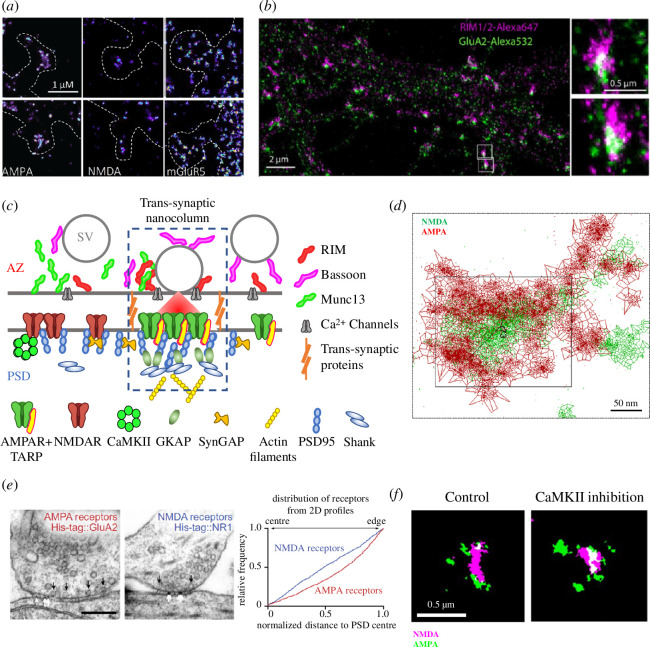
Synaptic nano-organization. (*a*) dSTORM images of endogenous glutamatergic receptors. From the left, AMPARs, NMDARs and mGluR5. Adapted from [92]. (*b*) Dual-colour dSTORM images of trans-synaptic nanocolumns. Images of GluA2-containing AMPAR labelled with Alexa 532 nm and regulating synaptic membrane exocytosis protein (RIM) labelled with Alexa 647 nm. Adapted from [93]. (*c*) Schematic of trans-synaptic nanocolumn molecular organization. Adapted from [94]. (*d*) Dual-colour dSTORM images of endogenous NR1-containing and GluA2-containing NMDARs and AMPARs in cultured hippocampal neurons. Adapted from [92]. (*e*) Immunogold labelling of AMPAR andl NMDAR with respect to glutamate release sites. Adapted from [95]. (*f*) Inhibition of CaMKII decreases the segregation of AMPAR and NMDAR sub-synaptic domains. Adapted from [96].

Studies in the 2000s questioned the existence of a sub-PSD organization within the PSD itself [84–86]. However, the study of such fine structures using traditional optical microscopy is impossible due to the ~300 nm diffraction limit of visible light [87]. This has been overcome in recent years by the application of super-resolution microscopy techniques [88–91]. In 2013, using direct Stochastic Optical Reconstruction Microscopy (dSTORM), universal Point Accumulation Imaging in Nanoscale Topography (uPAINT) and single particle tracking PhotoActivation Localization Microscopy (sptPALM), Nair *et al*. from our team for the first time demonstrated the presence of AMPAR nanodomains in synapses [89] (figure 1). Synapses typically contain 1–3 such AMPAR clusters, which are around 80 nm in size and contain 20–25 densely packed AMPARs [89]. MacGillavry *et al*. [88] as well as Fukata *et al*. [91] have simultaneously demonstrated that PSD95 forms clusters within the PSD that range from 80 to 150 nm in size, depending on the probes used for imaging.

Since AMPARs have a relatively low affinity for glutamate (Dissociation constant (Kd) in the hundreds of µM range) [97] and the concentration of glutamate rapidly drops with distance from the release site [98], the localization of AMPAR clusters facing release sites seemed a plausible novel mechanism to increase the probability of their activation [97], as it was demonstrated soon after [99]. Another important question relates to the co-organization of the various elements of the PSD. NMDARs typically form a single loose cluster located in the centre of the PSD and are surrounded by multiple AMPAR clusters [92,100] (figure 1*d*,*e*). NMDARs thus seem to not be co-organized with AMPARs and release sites, as also shown by electron microscopy [95]. This is consistent with the higher affinity of NMDARs for glutamate, imposing less constraint on their localization within the glutamate gradient. Conversely, mGluR5, which also has a high affinity for glutamate, has been found to be either evenly dispersed at the synaptic surface [92] or dynamically organized in peri-synaptic nanodomains that localize close to, but not in, the synapse [101]. The PSD is thus a highly complex environment organized at the nanoscale in different receptor domains that are organized in register with the pre-synaptic release machinery.

Indeed, in 2016, Tang *et al*. [99] demonstrated the existence of nano-clusters formed by the pre-synaptic scaffolding protein involved in synaptic vesicle organization and fusion—RIM1/2. They discovered, most interestingly, that these pre-synaptic clusters were aligned with the post-synaptic clusters of PSD95 [99]. This study identified the existence of trans-synaptic nanocolumns, which co-organize the pre-synaptic release machinery in register with post-synaptic AMPARs and improve the efficiency of synaptic transmission [87,88] (figure 1*b,c*). Interestingly, the recent developments in cryo-electron tomography have allowed the ultrastructural visualization of these protein nanodomains and trans-synaptic assemblies [102–108]. The burning question that thus arose was: what are the underlying molecular elements that bridge pre- and post-synaptic nanodomains?

Candidate adhesive nanocolumn organizers should possess at least one of three properties. First, they should participate in interactions that connect the pre- and post-synaptic membranes. Second, an organizer responsible for trans-synaptic alignment should display a localized pattern within the synaptic cleft that resembles the intrasynaptic nanodomains. Third, modifying their expression or disrupting their extracellular assembly should have an impact on the structure of the columns. While no protein family completely meets these expectations, many studies from different groups have identified promising candidates [109]. Different protein types have been proposed to participate in trans-synaptic complexes, including cell adhesion molecules (CAMs), secreted proteins, extracellular domains of post-synaptic receptors and pre-synaptic voltage-gated calcium channels (VGCCs) [109].

It has been proposed that the interaction between pre-synaptic adhesion molecule Neurexin (Nrxn) and post-synaptic Neuroligin (Nlg), which form a trans-synaptic bridge [110], could regulate this trans-synaptic alignment. We demonstrated that the expression of a C-terminal truncation mutant of Nlg-1 disrupts the alignment between pre-synaptic regulating synaptic membrane exocytosis protein (RIM) and post-synaptic AMPAR clusters, as well as impairs AMPAR-mediated synaptic transmission [93]. However, this does not provide direct evidence that the Nrxn–Nlg interaction is responsible for trans-synaptic alignment. Considering the vast functions of Nrxn–Nlg complexes in mediating synaptogenesis, a prolonged treatment could have unspecific effects other than affecting trans-synaptic alignment. Interestingly, it was recently shown that Nrxn3 controls excitatory synapse nano-organization in hippocampus and localizes discretely from Nrxn1 [111]. The aforementioned Nrxn and Nlg are members of the CAM family; however, the broad distribution of Nlg-1 over the PSD questions its involvement in nanocolumn alignment [109]. In contrast to Nlg-1, LRRTM2, a synaptogenic CAM, is localized in stable clusters on the post-synaptic membrane [112]. Both Nlg-1 and LRRTM2 bind to PSD95 on the post-synaptic side, and Nrxn pre-synaptically [113]. Ramsey *et al*.—using acute extracellular proteolysis of an engineered cleavage site in LRRTM2 to disrupt its extracellular interactions within seconds—demonstrated that LRRTM2 controls the positioning of AMPARs opposite to release sites in an acute manner. The misalignment led to a reduction in evoked AMPAR currents [113]. This established LRRTM2 as a strong candidate in maintaining trans-synaptic alignment of nanodomain clusters with the pre-synaptic machinery. Another candidate for molecularly linking the Active Zone (AZ) to synaptic cleft is Lipirin-α, which binds to RIM [114], as well as transmembrane protein tyrosine phosphatases of the leukocyte common antigen-related protein (LAR) family [115]. LAR proteins interact with various proteins present on the post-synaptic membrane, which are involved in regulating the development of excitatory synapses. These proteins include NGL/LRRC (netrin-G ligand/leucine-rich-repeat-containing), Slitrks (Slit- and Trk-like) and SALM (synaptic adhesion-like molecule) proteins. Therefore, LAR proteins have the potential to bridge molecules within the synaptic cleft [116–120]. Another candidate is EphB2, the post-synaptic receptor for pre-synaptic membrane-anchored ephrins, which is enriched towards the centre of the PSD [104]. Additionally, proteins displaying a broader distribution in the cleft could also help to align pre- and post-synaptic elements including adhesion molecules SynCAM-1 and N-Cadherin, which mark the post-synaptic edge [104,109]. The trans-synaptic adhesion complex involving N-cadherin at glutamatergic synapses is formed by N-cadherin proteins located in both the pre- and post-synaptic membranes [121], which interact in a *cis*- or *trans*-configuration [122,123]. Interestingly, N-Cadherin has also been suggested to directly bind to GluA2 subunits of AMPARs [124], which provides a direct coupling between the post-synaptic nano-organization of AMPARs and pre-synaptic organization. AMPARs have also been shown to directly bind to β3-integrin [125], a member of the integrin family of proteins that mediate cell-to-extracellular matrix (ECM) and cell-to-cell adhesion [126]. Importantly, the concept that AMPARs can themselves interact with ECM proteins or adhesion proteins has been greatly extended recently and probably also contributes to activity-dependent trapping of AMPARs at synaptic sites, as detailed below.

Soluble matrix molecules, pre- or post-synaptic receptors or channels could guide cleft organization through interactions of their extracellular domains with adhesion molecules or other partners [109]. An interesting candidate for mediating these interactions is neuronal pentraxins. These include two secreted proteins and a membrane-bound protein—the neuronal pentraxin receptor (NPR), expressed on the pre-synaptic membrane. NPR promotes the development of post-synaptic specializations, and intriguingly, its effect is regulated by neuronal activity [127] and the competitive antagonist of AMPARs, NBQX [128]. Secreted neuronal pentraxins bind to AMPARs, and through the interaction with pre-synaptic NPR, could bridge the trans-synaptic alignment [129]. The notion that pre-synaptic VGCCs play a role in affecting post-synaptic organization is intriguing. However, unlike most receptors, pre-synaptic VGCCs have a small extracellular domain, making it less likely for them to directly guide interactions within the synaptic cleft. Nonetheless, the presence of the glycosylphosphatidylinositol (GPI)-linked extracellular protein α2δ, which associates with VGCCs, may expand the scope of these interactions [130,131]. Other recently identified secreted proteins that have been proposed to regulate the anchoring of AMPARs and potentially trans-synaptic alignment are Noelin 1–3 [132,133]. Noelins are secreted proteins abundantly expressed in all brain regions that bind to the extracellular domains of GluAs and can induce their diffusion trapping [133]. Boudkkazi *et al*. [132] demonstrated that these homo- or heterotetramers, via an interaction with a network of proteins, anchor AMPAR complexes in post-synaptic membranes, and thus determine their density and organization. Impairment of this Noelin-based anchoring led to a cell-type specific decrease in surface AMPAR numbers as well as disrupted long-term synaptic plasticity in the CA3–CA1 pathway. Finally, secreted factors, including retrograde synaptic signals such as Wnt7a, Brain-Derived Neurotrophic Factor (BDNF) or endocannabinoids, could affect trans-synaptic alignment as well through post-translational modifications of interactors [109].

Altogether, a fascinating question thus becomes how regulation of synapse nanoscale landscape and trans-synaptic organization participates in LTP. We initially found that the number of AMPAR nanoclusters per spine scales with synapse size [89]. It was later established that LTP-inducing stimuli trigger the incorporation of additional nanomodules [134]. It remains to be understood how trans-synaptic nanomodules are organized and regulated and how additional AMPARs can be positioned or re-organized on the nanoscale during the early stages of LTP to efficiently participate in enhancing synaptic transmission.

## 4. The wealth of extrasynaptic diffusing surface AMPARs and their reversible stabilization at synapses

**Figure 2.**
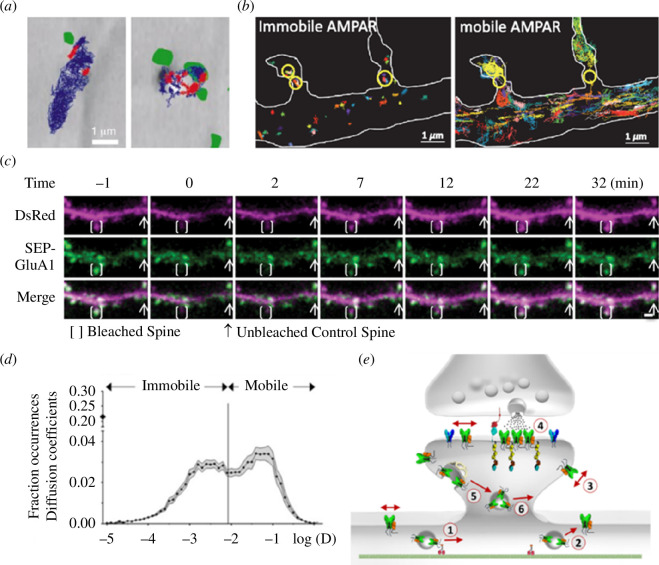
AMPAR surface mobility and overall AMPAR trafficking in and out synapses. (*a*) Representative trajectories of AMPARs labelled with latex beads in the proximity of pre-synaptic sites labelled with FM1-43 (green) in cultured hippocampal neurons. Diffusive movements in blue and confined movements in red. Adapted from [48]. (*b*) Images of high-resolution uPAINT of AMPAR surface mobility with individual AMPAR trajectories of immobile receptors (left) mostly present in nanoclusters (yellow circles) and mobile receptors (right), enriched outside nanoclusters. Adapted from [87]. (*c*) Representative multi-photon image *in vivo* of a bleached spine and fluorescent recovery in a layer 2/3 visual cortex dendrite. The cell is expressing cytosolic DsRed (magenta) and SEP-GluA1 (green). Adapted from [136]. (*d*) Distribution of AMPAR diffusion coefficients in spines. Approximately 30–50% of receptors are mobile with a *D* > 0.01 μm^2^ s^–1^. Adapted from [135]. (*e*) Model of AMPAR trafficking in and out of the PSD [1]. Newly synthesized receptors are transported inside the cell in vesicles [2]. Vesicles are exocytosed within the extrasynaptic compartment [3]. On the surface, AMPARs move randomly by Brownian diffusion and can be [4] reversibly stabilized through diffusion trapping in the PSD [5]. Diffusing receptors are internalized in the extrasynaptic compartment through clathrin-dependent endocytosis [6]. Endocytosed receptors can be recycled back through exocytosis. Adapted from [135].

**Figure 3.**
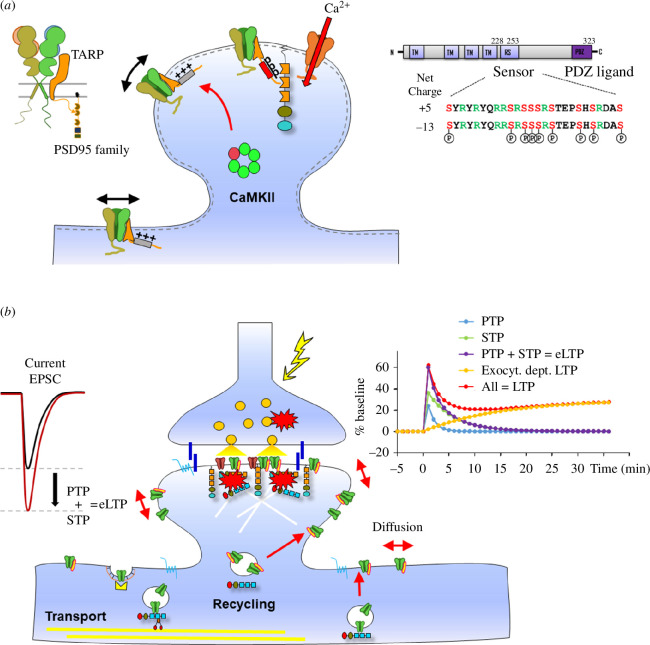
Model of the molecular mechanism of activity-dependent AMPAR diffusion trapping and role in early phases of LTP. (*a*) Left inset, TARP *γ*2 and *γ*8 AMPAR auxiliary subunits allow stabilization of AMPAR complexes to PSD95 intracellular scaffold thanks to the interaction of their intracellular c-terminus PDZ ligand with the PDZ domains of PSD95. Right inset, theC-terminal domain of TARP *γ*2 and *γ*8 contains an upstream Ser- and Arg-rich domain (‘Sensor’) and a terminal PDZ domain ligand. The Ser can be phosphorylated by CaMKII and PKC. Bottom, upon activity-triggered calcium influx through NMDAR, CaMKII translocates to the spine and phosphorylates the TARP Ser-rich domain. This allows the repulsion of the TARP c-terminus from the negatively charged plasma membrane, its extension into the cytoplasm and binding to PDZ domains of PSD95. This results in AMPAR immobilization (*b*). Upon pre-synaptic tetanic stimulation, an immediate (seconds to minutes) dual potentiation of synaptic strength occurs, owing to both pre-synaptic post-tetanic potentiation (PTP) and post-synaptic diffusion trapping of AMPARs (STP). Both phenomena occur on similar time-frames and cannot be distinguished based solely on kinetics, resulting in potentation of Excitatory Post Synaptic Currents (EPSC) early LTP (eLTP). In a secondary timeframe, intracellular AMPARs are exocytosed and incorporated in the plasma membrane to allow re-equilibration of the fraction of mobile AMPARs in synapses, leading to exocytosis-dependent sustained LTP. The sum of all these components forms LTP.

Given the importance of the nanoscale organization of AMPARs, their dynamics are bound to have important consequences. On average, 30–50% of AMPARs present at the cell surface are mobile, with a diffusion rate of over 0.01 μm^2^ s^–1^ [135] (figure 2). The diffusion of AMPARs within the PSD is remarkably rapid, influencing synaptic transmission within milliseconds. Specifically, AMPARs can traverse over 100 nm in just 10 ms. This mobility allows for about 30% of receptors to be exchanged within a 200 nm area in the same timeframe [46,137]. Such dynamics underscore AMPAR diffusion as a potent mechanism for modulating short-term synaptic plasticity and the turnover of desensitized receptors within the synapse, positioning it as a good mechanism to regulate short-term synaptic plasticity and recycling of desensitized receptors within the synapse [46,138]. This is particularly important for AMPARs that have a low affinity for glutamate [74] and are thus only activated within a small ~100 nm radius from the glutamate release site [46,97]. At excitatory synapses, receptor trafficking regulates activity-driven positioning of receptors in front of transmitter release sites, as reviewed in [24–27]. Receptors are more mobile in the extrasynaptic compartment and less so inside synapses, where they are trapped in saturable binding sites such as intracellularly stabilized scaffolds [87], and their mobility is hindered due to the high protein density [139–141]. This diffusional trapping step depends on the interaction between PSD95 and AMPAR auxiliary proteins [47]. Altogether, the regulation of AMPAR content during synaptic activity occurs through a three-step process. During synaptic potentiation, it includes AMPAR exocytosis at extrasynaptic sites, surface diffusion between extrasynaptic and synaptic sites and a diffusion trapping step within the PSD (figure 3*e*) [27]. Conversely, synaptic depression involves the diffusion of receptors out of synapses and endocytosis at extrasynaptic sites (figure 2*e*) [142]. AMPAR surface diffusion is tuned in response to physiological processes including the aforementioned long-term synaptic potentiation (LTP), when receptors are immobilized in synapses [45]. Conversely, both long-term synaptic depression induction [143] and corticosterone (stress hormone) administration, which change the set point for induction of LTP, trigger an increase in AMPAR mobility [136,144].

## 5. Mechanisms of AMPAR activity-dependent trapping at synaptic slots during LTP: the role of TARPs

The major signalling pathway for LTP induction is arguably the rapid increase in post-synaptic Ca^2+^ concentration following NMDAR and L-type calcium channel activation. This leads to the activation of downstream cascades, including the activation of CaMKII, which is associated with the NMDAR GluN2B subunit [145]. CaMKII then auto-phosphorylates, becoming independent of Ca^2+^ influx, and phosphorylates a set of substrates, including GluA1 and TARP AMPAR complex subunits, increasing among other things the conductance of the channel [53] and AMPAR trapping [50], which both enhance synaptic strength. The following steps of LTP and the changes in synaptic strength strongly rely on AMPAR trafficking. First, LTP induction, and in more detail, CaMKII activity, have been shown to induce surface AMPAR immobilization and trapping of receptors in synaptic sites [30,45,69], which may be independent of subunit composition [81]. As stated above, blocking the surface diffusion of AMPARs in the hippocampal CA1 region prevents the induction of LTP [69,70]. We proposed that this effect is owing to the existence of a large pool of extrasynaptic AMPAR [45,65,146] that can be rapidly recruited to synapses by diffusion trapping [27].

The first mechanism proposed to be involved in the trapping of receptors at synapses was through the interactions of AMPAR subunit C terminal domains (CTDs) with PDZ domain-containing proteins (PDZ is an initialism combining the first letters of the first three proteins discovered to share the domain—post synaptic density protein (PSD95), Drosophila disc large tumor suppressor (Dlg1), and zonula occludens-1 protein (zo-1)). This was stimulated by the cloning of various PDZ domain-containing proteins interacting selectively with AMPARs (reviewed in [13,23,147–149]). Several proteins have been suggested to directly interact with GluA CTDs and are necessary for synaptic plasticity and synaptic scaling. In particular, GluA2 and GluA3 share a consensus sequence, Ser–Val–Lys–Ile, at their C-terminus, through which Glutamate receptor-interacting protein (GRIP) family proteins (GRIP1, GRIP2 and AMPAR-binding protein) and PICK1 (Protein Interacting with C Kinase-1) interact whereas the GluA1 CTD contains a 4.1N and SAP97 binding site. In addition, LTP could be triggered by increased activity of the calcium-/calmodulin-dependent protein kinase II (CaMKII) [64]. This effect was not diminished by mutating the CaMKII phosphorylation site on GluA1 but was blocked by mutating a predicted PDZ domain interaction site. These results suggested that LTP and CaMKII activity drive AMPARs to synapses by a mechanism that requires the association between GluA1 and a PDZ domain protein [64]. This concept was recently reinforced by studies indicating that the CTDs of GluA1 and GluA2 are necessary and sufficient to drive NMDAR-dependent LTP and Long-Term Depression (LTD), respectively [150]. However, other studies have established that mutating the CTD protein interaction sites [81,151], or even complete CTD removal, does not prevent synaptic targeting of GluA1 or the expression of LTP. This was recently supported by Díaz-Alonso *et al*. [152]. Along this line, we found that mutating the PDZ binding site of GluA2 CTD does not modify AMPAR diffusion trapping [47]. While these results contradict those of Zhou *et al*. [150], it should be noted that in a follow-up study using knock-in mice where the CTD of GluA1 is replaced by that of GluA2, the authors observed that the requirement of GluA1 CTD during hippocampal LTP was age- and induction protocol-dependent [153], and therefore, could explain the discrepancy observed in previous studies as discussed in [149,154].

Interestingly, we recently demonstrated a key role of GluA1 CTD interaction with SAP97 and phosphorylation in AMPAR intracellular transport and its activity-dependent regulation [61,155]. Altogether, we would thus conclude that while GluA1 and GluA2 CTDs are likely to be differentially important for LTP expression, they probably do not participate in AMPAR diffusion trapping *per se* but rather in AMPAR intracellular transport, exocytosis and recycling.

A strong emphasis has, in contrast, been put on the role of AMPAR auxiliary protein-PSD95 family membrane-associated guanylate kinases (MAGUKs) in the diffusional trapping of AMPARs at the PSD. We first demonstrated that AMPARs are reversibly trapped at the PSD through the interaction between Stargazin—also known as TARP γ2—and PSD95 [47], and we then established the role of CaMKII-induced phosphorylation of γ2 on an activity-dependent increase in AMPAR trapping [45]. Finally, we demonstrated that AMPAR diffusion in and out of synaptic sites is a requirement during synaptic plasticity [69,70,135]. These elements further reinforce the motivation to understand the molecular mechanisms for activity-dependent AMPAR trapping at synapses.

γ2 is the prototypical AMPAR auxiliary subunit. Its expression is notably abundant in the cerebellum, where it facilitates the delivery of AMPARs to the plasma membrane of cerebellar granule cells, while it exhibits a modest expression in the hippocampus [156–158]. Additionally, γ2 has also been implicated in several other AMPAR-related functions such as surface diffusion [45,47,159,160], stabilization [161], endocytosis [162] and synaptic targeting of AMPARs [156,163,164]. γ2 has consequently been highly involved during synaptic plasticity [165,166] and synaptic scaling [167,168].

γ2 contains a C-terminal canonical PDZ-binding motif, which has been shown to interact particularly with PSD95, but also PSD93 [156,169], in a phosphorylation-dependent manner [160,170,171]. Consequently, γ2 and PSD95 interaction has been proposed to mediate AMPAR synaptic abundance by regulating receptor diffusion trapping at synaptic sites from extrasynaptic receptors [47,164,172]. Indeed, deletion of the γ2 PDZ-binding motif destabilizes synaptic AMPARs, as observed by their increased mobility at PSD sites and decreased AMPAR-mediated synaptic currents [47,164]. Note that the PDZ-binding motif is not the only motif at the TARPs CTD responsible for the interaction with PSD95. In addition to the PDZ-binding motif, type-I TARPs CTD display three additional conserved motifs—the Ser-rich motif, Arg-rich motif and three aromatic residues—containing hydrophobic motifs (φ1/2/3) [173]. The positively charged stretch of conserved Arg residues mediates TARPs–CTD interaction with the negatively charged phospholipids of lipid membranes via electrostatic interactions [160,174], crucial during AMPAR-mediated plasticity [173], whereas some of the Ser residues that compose the Ser-rich motif have been implicated in the regulation of TARPs-dependent AMPAR-mediated plasticity via PSD95 interaction, as further discussed below.

Recently, Zeng *et al*. observed that the γ2 CTD undergoes liquid–liquid phase separation with PSD95 [173], a characteristic of several PSD proteins [96,175,176]. Interestingly, all four domains of TARPs CTD were shown to influence the formation of the liquid–liquid phase separation with PSD95, while the aromatic residues containing hydrophobic motifs exert a moderate-to-strong effect on the formation of the liquid–liquid phase separation, both PDZ-binding motif and the Ser- and Arg-rich motifs are crucial in the formation of this lipid-like droplet between γ2 and PSD95 [173]. In neurons, the two first PSD95 PDZ domains (PDZ1 and PDZ2) have been suggested as the preferential binding sites of γ2-containing AMPARs [164,177]. It is worth highlighting that γ2 CTD and PSD95 binding affinity is one of the strongest PSD95 PDZ1/PDZ2–target interactions ever reported [173]. Interestingly, Zeng *et al*. [173] observed that the PDZ-binding motif of γ2 binds with the PSD95 PDZ2 domain, and the Arg-rich motif binds to the PSD95 PDZ1 domain. Thus, the entire C-terminal tail of γ2 is required for binding to PSD95, resulting in a highly specific and multivalent γ2/PSD95 complex. However, phosphorylation of the Ser-rich motif causes elongation of the γ2 CTD into the cytosol, which shifts the γ2 CTD binding to the deepest- and highest-affinity PDZ2/PDZ3 domains of PSD95 [160]. Altogether, the multivalent interaction between TARPs CTD and PSD95 is essential for trapping and stabilization of TARP-containing AMPARs at PSD sites, and consequently, for synaptic transmission.

Phosphorylation/dephosphorylation of the γ2 PDZ-binding motif and subsequent binding to PSD95 has been associated with synaptic plasticity. In dissociated hippocampal neurons, phosphorylation of Thr321 in the PDZ-binding motif of γ2 by PKA promotes synaptic targeting of γ2 and is required during chemical LTP induction, whereas LTD requires dephosphorylation of Thr321 of γ2 by mitogen-activated protein kinases (MAPKs) [165]. The most interesting target is, however, the phosphorylation at the conserved Ser-rich motif. Phosphorylation of the Ser-rich motif prevents binding of the γ2 CTD to the lipid bilayer by neutralizing the positively charged Arg-rich motif. Consequently, phosphorylation of the Ser-rich motif induces effective extension of the CTD into the cytosol, which favours γ2 interaction with PSD95; the opposite is observed during dephosphorylation [160,174]. NMDAR-dependent synaptic activity was shown to regulate both phosphorylation and dephosphorylation of the γ2 Ser-rich domain [178]. NMDAR-dependent LTP triggers CaMKII-dependent phosphorylation of γ2 [179,180], which in turn immobilizes γ2-containing AMPARs at synaptic sites [45]. Also, hippocampal activation of ghrelin was shown to trigger PKC-dependent phosphorylation of γ2 Ser-rich domain in an activity-dependent manner during LTP [181]. On the other hand, NMDAR-dependent LTD promotes γ2 dephosphorylation by phosphatase 1 (PP1)—downstream of calcium-/calmodulin-dependent protein phosphatase 2B (PP2B)/calcineurin [180]—and subsequent association with adaptor proteins to induce clathrin-dependent AMPAR endocytosis [162]. Consistently, cerebellar LTD at parallel fibre (PF) onto Purkinje cell synapses requires calcineurin-mediated dephosphorylation of γ2 Ser-rich domain [166].

In addition to synaptic plasticity, γ2 is also involved in AMPAR-mediated synaptic scaling (or homeostatic scaling), a form of homeostatic plasticity in which neurons adjust their synaptic strength in response to chronic alterations in the network activity by regulating the number of synaptic receptors [182]. Tetrodotoxin-induced synaptic upscaling triggers AMPAR synaptic delivery that is dependent on phosphorylation of γ2 Ser-rich domain [167]. In contrast, synaptic downscaling triggers dephosphorylation of γ2 Ser-rich domain, which increases γ2 and AMPAR mobility at the plasma membrane and AMPAR endocytosis [168].

A topic that has raised a lot of controversy—and no consensus has been achieved to date—is whether or not auxiliary subunits dissociate from the receptor at the plasma membrane. Upon AMPA-induced AMPAR internalization, Tomita *et al*. [161] observed that γ2 and γ3 internalized levels were unaffected by AMPA treatment, while AMPARs were heavily internalized. Moreover, AMPA-induced internalization led to a decrease in AMPAR and γ3 association at the surface, which suggested a possible dissociation of TARPs from the AMPAR complex. These observations were later backed up by the observation that glutamate induced a transient dissociation of γ2 from AMPAR complexes [159,183]. However, the veracity of this dissociation has been questioned by others’ work [184–186] in functional [187,188] and structural studies [189–192].

Importantly, TARP γ8, which is quite homologous to γ2, is actually the most abundant TARP subtype in the hippocampus [158,193]. γ8 mostly regulates AMPAR basal transmission in CA1 neurons. Loss of γ8 leads to a nearly complete depletion of extrasynaptic AMPAR-mediated currents, but only to a mild reduction of the synaptic AMPAR-mediated currents [194]. As mentioned above, LTP requires AMPAR recruitment and trapping at synapses. Consistently, loss of γ8 drastically impairs LTP at CA1 synapses [194] and dentate gyrus medial perforant path synapses [195], but not LTD [194]. γ8 CTD is subject to phosphorylation [196,197], of which the γ8 CaMKII-dependent phosphorylation sites Ser277 and Ser281 at the Ser-rich motif were shown to mediate hippocampal LTP and learning and memory [197]. Compared to other TARPs, γ8 has a unique long CTD containing a Pro/Ala-rich domain that binds to calcineurin/PP2B [198]. It is therefore possible that this interaction mediates trafficking and phosphorylation of AMPARs. γ8 PDZ-binding motif was shown to regulate AMPAR-mediated basal synaptic transmission, but not synaptic plasticity, i.e. LTP [199]. Conversely, Sheng *et al*. [200] observed that γ8 PDZ-binding motif was necessary for LTP at CA1 synapses, but not for phosphorylation of the γ8 Ser-rich motif. However, the striking difference between Sheng *et al*. and previous studies is most likely owing to different experimental strategies, as Sheng *et al*. relied on overexpression of GluA1 and γ8 in triple-floxed Gria1-3 mice. Therefore, the authors hypothesized that in the case of GluA1 homomeric AMPARs, γ8 PDZ interaction, but not Ser-rich motif phosphorylation, was necessary for synaptic targeting, and consequently LTP induction [200]. While γ8 PDZ-binding motif is crucial for the synaptic targeting and transmission of GluA1 homomeric AMPARs, this is not necessarily valid for other GluA2-containing AMPARs [151]. It is interesting to note that the Arg-rich motif of γ2 and γ8 was recently shown to mediate PSD95 interaction and be required for hippocampal LTP [173].

Despite the large body of evidence involving γ2 and γ8 in diffusional trapping of AMPAR at synapses and the role of their C terminal phosphorylation, the overall mechanism of action might not be as simple as previously thought. Recent work by Zeng *et al*. [173] in particular reports data that are hard to reconcile with the concept of TARP-dependent AMPAR diffusion trapping. Zeng *et al.*'s study [173] not only demonstrates that the C-tails of γ2 and γ8 bind PSD95 through their PDZ ligand and through the upstream Arg-rich motif, but indicates that changing the charge of this Arg-rich motif in γ2, such as occurs during phosphorylation on the neighbouring serines, induces increased mobility of the C-tail of γ2 in artificial bilayers and phase separation of the γ2 C-tail/PSD95. In addition, introducing this arginine to alanine mutation in γ8 fused in tandem with GluA1 massively decreases Excitatory Post Synaptic Current (EPSC) size and prevents LTP in a model of GluA1-γ8 tandem expression in a triple GluA1,2,3 knock-out. These experiments are at variance with previous data showing that similar Arg to Ala or Ser to Asp mutations increase γ2 binding to PSD95, AMPAR synaptic trapping, mEPSCs and synaptic currents [45,160,180]. We have no explanation at this stage for this discrepancy and are thus left with no robust molecular model for the activity-dependent diffusion trapping of AMPAR. One hypothesis could be that there is a subtle interplay between the functional roles of γ2- and γ8-containing AMPARs that have not yet been captured. Along this line, we have recently shown using genetic code expansion that γ2 and γ8 display very different subcellular localizations, γ2 being nearly exclusively synaptic while γ8 is present both synaptically and extrasynaptically [201], as previously suggested [193]. This suggests that both TARPs may play differential roles in trapping AMPARs during LTP.

## 6. Mechanisms and necessity for AMPAR activity-dependent trapping at synaptic slots during LTP: emerging concepts and outlook

While many reports have demonstrated that AMPAR lateral diffusion can be tuned by neuronal activity or hormones [30,31,45,46,48,69,70,136,144,202,203], it has been difficult to reciprocally demonstrate that AMPAR diffusion trapping is necessary for LTP. Indeed, contrary to the classical genetic or pharmacological means used to demonstrate the implication of a given protein in a process, receptor surface diffusion is less amenable to manipulations. Fifteen years ago, we developed the approach of surface receptor cross-linking in neurons to specifically prevent their surface diffusion. This approach has been extensively used in non-neuronal cells to, for example, mimic multivalent antigen binding to antigen receptors [204]. However, in most cases, the application of a multivalent ligand to a surface receptor induces its rapid capping on one pole of the cell. Some autoantibodies to AMPAR found in auto-immune diseases have been shown to induce AMPAR internalization in neurons and switch in subunits, which may be directly related to disease progression [205]. Importantly, we did not observe this phenomenon in neurons with most lab-made antibodies. In contrast, we observed that multivalent ligand-induced cross-linking of surface neurotransmitter receptors triggered their rapid immobilization without associated noticeable internalization, surface re-organization, or change in basal synaptic transmission [46,69,206,207]. We believe this is due to the fact that in neurons, at rest, a sizeable fraction of surface receptors is already immobile (e.g. ~50% of AMPARs are immobile at rest in neurons). Thus, upon the addition of a cross-linker, mobile receptors are rapidly immobilized through linking with a neighbouring immobile partner, preventing the formation of a cap and massive re-distribution of the receptors. This approach allowed us to explore the functional role of the surface diffusion of a variety of receptors in diverse functional settings.

First and foremost, using either antibody to native receptors or avidin derivatives for biotinylated receptors, we could demonstrate the necessity for AMPAR surface mobility in the expression of early LTP [46,69,70,207] as well as in fear conditioning memory formation [69,70], in cortical remapping and adaptive behaviours during sensory experience [207] or recently in memory consolidation [206]. The most conservative explanation for the ability of surface AMPAR cross-linking to block these LTP-associated processes is that AMPAR diffusion trapping is necessary for their activity-dependent accumulation at synaptic sites. Alternatively, LTP could be owing to local AMPAR reorganization on a nanoscale level that could alter their positioning relative to glutamate release sites, thereby influencing activity-dependent changes in synaptic transmission. AMPAR cross-link could block this reorganization and thereby block LTP. However, none of these have been yet observed.

Using the cross-linking approach, we could also identify a new modality of regulation of high-frequency dependent short-term plasticity by the rapid exchange of desensitized AMPARs by naive ones [46]. A similar mechanism was found at play for kainate receptors [208] and, to some extent, for GABA receptors [209]. Others found that the surface dynamics of GluN2B-NMDA receptors control plasticity of maturing glutamate synapses [210], that surface diffusion of astrocytic glutamate transporters shapes synaptic transmission [211] and that aquaporin-4 surface trafficking regulates astrocytic process motility and synaptic activity [212]. It is particularly interesting to note that many neurotransmitter receptor types are organized in nanodomains in the PSD (or subsynaptic domains—SSD) (e.g. [88,89,213,214]) and not diffusively distributed in the PSD as previously thought. This nanoscale organization, coupled with receptor dynamic exchange between domains, is bound to have profound impacts on synaptic physiology, particularly for receptors having a low affinity for their ligand, as explained above. Along this line, it was recently shown that a pre-synaptically bound synthetic synaptic cross-linker protein binding to AMPAR could restore normal glutamatergic synapse function in a model of Alzheimer’s disease [215].

In the future, it will be interesting to expand the approach of cross-linking to the immobilization of AMPAR auxiliary proteins. This will require using genetic code expansion to incorporate unnatural amino acids that can be a platform for cross-linking as the extracellular domains of AMPAR auxiliary proteins are usually not accessible to bulky multimeric ligands [201]. This will allow testing the putative existence of AMPAR–TARPs dissociation at the plasma membrane. Complementary approaches include the capacity to manipulate intracellular scaffold protein dynamics and interaction with partners using light [83,216].

Regarding the mechanism of AMPAR activity-dependent diffusion trapping at synapses, while most studies have so far concentrated on the role of CaMKII-induced phosphorylation of TARPs or other AMPAR auxiliary proteins such as Shisa6, and consecutively increased binding to PSD95, several new results may call for revisiting this theory. Recently, Tullis *et al*. [217] demonstrated that LTP induction is provoked by structural rather than enzymatic functions of CaMKII. Directly activating the structural function of CaMKII was sufficient to elicit robust LTP, even when enzymatic CaMKII activity was blocked. How AMPAR accumulation could be driven in this case remains to be elucidated. The recent emergence of the concept of liquid–liquid phase separation (LLPS) of interacting synaptic proteins [176] might be a clue to this process. Along this line, another interesting piece to this puzzle is the synaptic Ras/Rap GTPase-activating protein (SynGAP) that plays substantial, albeit still elusive, roles in synaptic function [218] through both its GTPase activity and capacity to bind the PDZ domain of PSD95—the so-called ‘slots’. Recent [219] and older [220–222] work have suggested that activity-dependent phosphorylation of SynGAP induces its unbinding from PSD95 and dispersion away from synapses, allowing binding of new AMPAR–TARP complexes. SynGap can form LLPS with PSD95 and TARPs that are regulated by phosphorylation [173]. Importantly, loss-of-function mutations in the *SYNGAP1* gene may account for up to 1% of genetically based intellectual disabilities. Thus, SynGAP may emerge as a key activity-dependent molecular switch for AMPAR diffusion trapping.

The growing interest in the role of AMPAR extracellular N-terminal domains (NTDs) presents an intriguing development [129,151,223]. It has been suggested that AMPAR NTDs play a crucial role in regulating the stabilization of AMPARs at synapses; although research in this area of GluA biology is still in its early stages, this promises to reveal fascinating insights. A role for the AMPAR NTD in synaptic recruitment, anchoring and LTP was first documented in 2007 and then refined in 2017 [129,223,224] and has since been developed [151,152,225]. Indeed, several extracellular or adhesion proteins have been shown to bind GluA subunits such as pentraxins [129], cadherins [124] or noelin [133], and could act as extracellular diffusion trapping scaffolds/slots. How neuronal activity could regulate these N-terminal interactions, however, remains to be determined.

To conclude, determining the precise molecular mechanisms underlying the various forms of activity-dependent changes in synaptic efficacy is fundamental given their supposed role in memory formation and storage, as well as the vast number of brain diseases implicated in their dysfunction. In these processes, we believe that activity-regulated AMPAR diffusion trapping is instrumental in regulating AMPAR functional access to vesicularly released glutamate. This occurs through the control of their entry and exit from the PSD, control of their stabilization in the PSD through binding to intracellular or extracellular scaffold elements, and control of their nanoscale positioning in front of glutamate release sites. Integrating the conceptual frameworks of LLPS, slots, and diffusion trapping may allow a better understanding of activity-dependent changes in synaptic strength. Identifying the exact molecular elements and signalling pathways involved in each of these steps is important, not only for a full understanding of synaptic function but also to develop new leads for possible cures of synapse-associated brain diseases.

## Data Availability

This article has no additional data.

## References

[1] Bliss TV , Lomo T . 1973 Long-lasting potentiation of synaptic transmission in the dentate area of the anaesthetized rabbit following stimulation of the perforant path. J. Physiol. **232** , 331–356. (10.1113/jphysiol.1973.sp010273) 4727084 PMC1350458

[2] Bear MF , Malenka RC . 1994 Synaptic plasticity: LTP and LTD. Curr. Opin. Neurobiol. **4** , 389–399. (10.1016/0959-4388(94)90101-5) 7919934

[3] Enoki R , Hu YL , Hamilton D , Fine A . 2009 Expression of long-term plasticity at individual synapses in hippocampus is graded, bidirectional, and mainly presynaptic: optical quantal analysis. Neuron **62** , 242–253. (10.1016/j.neuron.2009.02.026) 19409269

[4] Lisman J . 2003 Long-term potentiation: outstanding questions and attempted synthesis. Phil. Trans. R. Soc. Lond. B **358** , 829–842. (10.1098/rstb.2002.1242) 12740130 PMC1693147

[5] Bliss TV , Collingridge GL . 1993 A synaptic model of memory: long-term potentiation in the hippocampus. Nature **361** , 31–39. (10.1038/361031a0) 8421494

[6] Kerchner GA , Nicoll RA . 2008 Silent synapses and the emergence of a postsynaptic mechanism for LTP. Nat. Rev. Neurosci. **9** , 813–825. (10.1038/nrn2501) 18854855 PMC2819160

[7] Isaac JT , Nicoll RA , Malenka RC . 1995 Evidence for silent synapses: implications for the expression of LTP. Neuron **15** , 427–434. (10.1016/0896-6273(95)90046-2) 7646894

[8] Liao D , Hessler NA , Malinow R . 1995 Activation of postsynaptically silent synapses during pairing-induced LTP in CA1 region of hippocampal slice. Nature **375** , 400–404. (10.1038/375400a0) 7760933

[9] Nicoll RA , Malenka RC . 1999 Expression mechanisms underlying NMDA receptor-dependent long-term potentiation. Ann. N. Y. Acad. Sci. **868** , 515–525. (10.1111/j.1749-6632.1999.tb11320.x) 10414328

[10] Collingridge GL , Kehl SJ , McLennan H . 1983 The antagonism of amino acid-induced excitations of rat hippocampal CA1 neurones in vitro. J. Physiol. **334** , 19–31. (10.1113/jphysiol.1983.sp014477) 6134823 PMC1197297

[11] Banke TG , Bowie D , Lee H , Huganir RL , Schousboe A , Traynelis SF . 2000 Control of GluR1 AMPA receptor function by cAMP-dependent protein kinase. J. Neurosci. **20** , 89–102. (10.1523/JNEUROSCI.20-01-00089.2000) 10627585 PMC6774102

[12] Derkach V , Barria A , Soderling TR . 1999 Ca^2+^/calmodulin-kinase II enhances channel conductance of α-amino-3-hydroxy-5-methyl-4-isoxazolepropionate type glutamate receptors. Proc. Natl Acad. Sci. USA **96** , 3269–3274. (10.1073/pnas.96.6.3269) 10077673 PMC15931

[13] Scannevin RH , Huganir RL . 2000 Postsynaptic organization and regulation of excitatory synapses. Nat. Rev. Neurosci. **1** , 133–141. (10.1038/35039075) 11252776

[14] Lynch G , Baudry M . 1984 The biochemistry of memory: a new and specific hypothesis. Science **224** , 1057–1063. (10.1126/science.6144182) 6144182

[15] Madison DV , Malenka RC , Nicoll RA . 1991 Mechanisms underlying long-term potentiation of synaptic transmission. Annu. Rev. Neurosci. **14** , 379–397. (10.1146/annurev.ne.14.030191.002115) 1851607

[16] Bredt DS , Nicoll RA . 2003 AMPA receptor trafficking at excitatory synapses. Neuron **40** , 361–379. (10.1016/s0896-6273(03)00640-8) 14556714

[17] Carroll RC , Beattie EC , von Zastrow M , Malenka RC . 2001 Role of AMPA receptor endocytosis in synaptic plasticity. Nat. Rev. Neurosci. **2** , 315–324. (10.1038/35072500) 11331915

[18] Collingridge GL , Isaac JTR , Wang YT . 2004 Receptor trafficking and synaptic plasticity. Nat. Rev. Neurosci. **5** , 952–962. (10.1038/nrn1556) 15550950

[19] Lüthi A , Chittajallu R , Duprat F , Palmer MJ , Benke TA , Kidd FL , Henley JM , Isaac JT , Collingridge GL . 1999 Hippocampal LTD expression involves a pool of AMPARs regulated by the NSF–GluR2 interaction. Neuron **24** , 389–399. (10.1016/s0896-6273(00)80852-1) 10571232

[20] Malenka RC , Nicoll RA . 1999 Long-term potentiation—a decade of progress? Science **285** , 1870–1874. (10.1126/science.285.5435.1870) 10489359

[21] Mammen AL , Kameyama K , Roche KW , Huganir RL . 1997 Phosphorylation of the α-amino-3-hydroxy-5-methylisoxazole4-propionic acid receptor GluR1 subunit by calcium/calmodulin-dependent kinase II. J. Biol. Chem. **272** , 32528–32533. (10.1074/jbc.272.51.32528) 9405465

[22] Nishimune A , Isaac JTR , Molnar E , Noel J , Nash SR , Tagaya M , Collingridge GL , Nakanishi S , Henley JM . 1998 NSF binding to GluR2 regulates synaptic transmission. Neuron **21** , 87–97. (10.1016/s0896-6273(00)80517-6) 9697854

[23] Song I , Huganir RL . 2002 Regulation of AMPA receptors during synaptic plasticity. Trends Neurosci. **25** , 578–588. (10.1016/s0166-2236(02)02270-1) 12392933

[24] Anggono V , Huganir RL . 2012 Regulation of AMPA receptor trafficking and synaptic plasticity. Curr. Opin. Neurobiol. **22** , 461–469. (10.1016/j.conb.2011.12.006) 22217700 PMC3392447

[25] Bard L , Groc L . 2011 Glutamate receptor dynamics and protein interaction: lessons from the NMDA receptor. Mol. Cell. Neurosci. **48** , 298–307. (10.1016/j.mcn.2011.05.009) 21640188

[26] Lisman J , Raghavachari S . 2006 A unified model of the presynaptic and postsynaptic changes during LTP at CA1 synapses. Sci. STKE **2006** , re11. (10.1126/stke.3562006re11) 17033044

[27] Opazo P , Choquet D . 2011 A three-step model for the synaptic recruitment of AMPA receptors. Mol. Cell. Neurosci. **46** , 1–8. (10.1016/j.mcn.2010.08.014) 20817097

[28] Groc L , Choquet D . 2020 Linking glutamate receptor movements and synapse function. Science **368** , eaay4631. (10.1126/science.aay4631) 32527803

[29] Park M , Penick EC , Edwards JG , Kauer JA , Ehlers MD . 2004 Recycling endosomes supply AMPA receptors for LTP. Science **305** , 1972–1975. (10.1126/science.1102026) 15448273

[30] Petrini EM , Lu J , Cognet L , Lounis B , Ehlers MD , Choquet D . 2009 Endocytic trafficking and recycling maintain a pool of mobile surface AMPA receptors required for synaptic potentiation. Neuron **63** , 92–105. (10.1016/j.neuron.2009.05.025) 19607795 PMC2847611

[31] Makino H , Malinow R . 2009 AMPA receptor incorporation into synapses during LTP: the role of lateral movement and exocytosis. Neuron **64** , 381–390. (10.1016/j.neuron.2009.08.035) 19914186 PMC2999463

[32] Shi SH , Hayashi Y , Petralia RS , Zaman SH , Wenthold RJ , Svoboda K , Malinow R . 1999 Rapid spine delivery and redistribution of AMPA receptors after synaptic NMDA receptor activation. Science **284** , 1811–1816. (10.1126/science.284.5421.1811) 10364548

[33] Rumpel S , LeDoux J , Zador A , Malinow R . 2005 Postsynaptic receptor trafficking underlying a form of associative learning. Science **308** , 83–88. (10.1126/science.1103944) 15746389

[34] Graves AR *et al* . 2021 Visualizing synaptic plasticity in vivo by large-scale imaging of endogenous AMPA receptors. Elife **10** , e66809. (10.7554/eLife.66809) 34658338 PMC8616579

[35] Roth RH , Cudmore RH , Tan HL , Hong I , Zhang Y , Huganir RL . 2020 Cortical synaptic AMPA receptor plasticity during motor learning. Neuron **105** , 895–908. (10.1016/j.neuron.2019.12.005) 31901303 PMC7060107

[36] Yudowski GA , Puthenveedu MA , Leonoudakis D , Panicker S , Thorn KS , Beattie EC , von Zastrow M . 2007 Real-time imaging of discrete exocytic events mediating surface delivery of AMPA receptors. J. Neurosci. **27** , 11112–11121. (10.1523/JNEUROSCI.2465-07.2007) 17928453 PMC3249441

[37] Yudowski GA , Puthenveedu MA , von Zastrow M . 2006 Distinct modes of regulated receptor insertion to the somatodendritic plasma membrane. Nat. Neurosci. **9** , 622–627. (10.1038/nn1679) 16604070

[38] Lin DT , Makino Y , Sharma K , Hayashi T , Neve R , Takamiya K , Huganir RL . 2009 Regulation of AMPA receptor extrasynaptic insertion by 4.1N, phosphorylation and palmitoylation. Nat. Neurosci. **12** , 879–887. (10.1038/nn.2351) 19503082 PMC2712131

[39] Patterson MA , Szatmari EM , Yasuda R . 2010 AMPA receptors are exocytosed in stimulated spines and adjacent dendrites in a Ras-ERK-dependent manner during long-term potentiation. Proc. Natl Acad. Sci. USA **107** , 15951–15956. (10.1073/pnas.0913875107) 20733080 PMC2936631

[40] Wu D , Bacaj T , Morishita W , Goswami D , Arendt KL , Xu W , Chen L , Malenka RC , Südhof TC . 2017 Postsynaptic synaptotagmins mediate AMPA receptor exocytosis during LTP. Nature **544** , 316–321. (10.1038/nature21720) 28355182 PMC5734942

[41] Jurado S , Goswami D , Zhang Y , Molina AJM , Südhof TC , Malenka RC . 2013 LTP requires a unique postsynaptic SNARE fusion machinery. Neuron **77** , 542–558. (10.1016/j.neuron.2012.11.029) 23395379 PMC3569727

[42] Ahmad M , Polepalli JS , Goswami D , Yang X , Kaeser-Woo YJ , Südhof TC , Malenka RC . 2012 Postsynaptic complexin controls AMPA receptor exocytosis during LTP. Neuron **73** , 260–267. (10.1016/j.neuron.2011.11.020) 22284181 PMC3269030

[43] Lledo PM , Zhang X , Südhof TC , Malenka RC , Nicoll RA . 1998 Postsynaptic membrane fusion and long-term potentiation. Science **279** , 399–403. (10.1126/science.279.5349.399) 9430593

[44] Passafaro M , Piëch V , Sheng M . 2001 Subunit-specific temporal and spatial patterns of AMPA receptor exocytosis in hippocampal neurons. Nat. Neurosci. **4** , 917–926. (10.1038/nn0901-917) 11528423

[45] Opazo P , Labrecque S , Tigaret CM , Frouin A , Wiseman PW , De Koninck P , Choquet D . 2010 CaMKII triggers the diffusional trapping of surface AMPARs through phosphorylation of stargazin. Neuron **67** , 239–252. (10.1016/j.neuron.2010.06.007) 20670832

[46] Heine M , Groc L , Frischknecht R , Béïque JC , Lounis B , Rumbaugh G , Huganir RL , Cognet L , Choquet D . 2008 Surface mobility of postsynaptic AMPARs tunes synaptic transmission. Science **320** , 201–205. (10.1126/science.1152089) 18403705 PMC2715948

[47] Bats C , Groc L , Choquet D . 2007 The interaction between stargazin and PSD-95 regulates AMPA receptor surface trafficking. Neuron **53** , 719–734. (10.1016/j.neuron.2007.01.030) 17329211

[48] Borgdorff AJ , Choquet D . 2002 Regulation of AMPA receptor lateral movements. Nature **417** , 649–653. (10.1038/nature00780) 12050666

[49] Ashby MC , Maier SR , Nishimune A , Henley JM . 2006 Lateral diffusion drives constitutive exchange of AMPA receptors at dendritic spines and is regulated by spine morphology. J. Neurosci. **26** , 7046–7055. (10.1523/JNEUROSCI.1235-06.2006) 16807334 PMC6673929

[50] Choquet D . 2018 Linking nanoscale dynamics of AMPA receptor organization to plasticity of excitatory synapses and learning. J. Neurosci. **38** , 9318–9329. (10.1523/JNEUROSCI.2119-18.2018) 30381423 PMC6705996

[51] Benke TA , Lüthi A , Isaac JTR , Collingridge GL . 1998 Modulation of AMPA receptor unitary conductance by synaptic activity. Nature **393** , 793–797. (10.1038/31709) 9655394

[52] Huganir RL , Nicoll RA . 2013 AMPARs and synaptic plasticity: the last 25 years. Neuron **80** , 704–717. (10.1016/j.neuron.2013.10.025) 24183021 PMC4195488

[53] Kristensen AS , Jenkins MA , Banke TG , Schousboe A , Makino Y , Johnson RC , Huganir R , Traynelis SF . 2011 Mechanism of Ca^2+^/calmodulin-dependent kinase II regulation of AMPA receptor gating. Nat. Neurosci. **14** , 727–735. (10.1038/nn.2804) 21516102 PMC3102786

[54] Barria A , Muller D , Derkach V , Griffith LC , Soderling TR . 1997 Regulatory phosphorylation of AMPA-type glutamate receptors by CaM-KII during long-term potentiation. Science **276** , 2042–2045. (10.1126/science.276.5321.2042) 9197267

[55] Perszyk RE *et al* . 2021 The negative allosteric modulator EU1794-4 Reduces single-channel conductance and Ca^2+^ permeability of GluN1/GluN2A N-methyl-d-aspartate receptors. Mol. Pharmacol. **99** , 399–411. (10.1124/molpharm.120.000218) 33688039 PMC8058507

[56] Lüthi A , Wikström MA , Palmer MJ , Matthews P , Benke TA , Isaac JTR , Collingridge GL . 2004 Bi-directional modulation of AMPA receptor unitary conductance by synaptic activity. BMC Neurosci. **5** , 44. (10.1186/1471-2202-5-44) 15538948 PMC535344

[57] Park P , Kang H , Sanderson TM , Bortolotto ZA , Georgiou J , Zhuo M , Kaang BK , Collingridge GL . 2019 On the role of calcium-permeable AMPARs in long-term potentiation and synaptic tagging in the rodent hippocampus. Front. Synaptic Neurosci. **11** , 4. (10.3389/fnsyn.2019.00004) 30923499 PMC6426746

[58] Babiec WE , Guglietta R , O’Dell TJ . 2016 Basal levels of AMPA receptor GluA1 subunit phosphorylation at threonine 840 and serine 845 in hippocampal neurons. Learn. Mem. **23** , 127–133. (10.1101/lm.040675.115) 26980779 PMC4793196

[59] Park P *et al* . 2021 PKA drives an increase in AMPA receptor unitary conductance during LTP in the hippocampus. Nat. Commun. **12** , 413. (10.1038/s41467-020-20523-3) 33462202 PMC7814032

[60] Cull-Candy SG , Farrant M . 2021 Ca^2+^ -permeable AMPA receptors and their auxiliary subunits in synaptic plasticity and disease. J. Physiol. **599** , 2655–2671. (10.1113/JP279029) 33533533 PMC8436767

[61] Bonnet C , Charpentier J , Retailleau N , Choquet D , Coussen F . 2023 Regulation of different phases of AMPA receptor intracellular transport by 4.1N and SAP97. Elife **12** , e85609. (10.7554/eLife.85609) 37079350 PMC10118384

[62] Malinow R , Malenka RC . 2002 AMPA receptor trafficking and synaptic plasticity. Annu. Rev. Neurosci. **25** , 103–126. (10.1146/annurev.neuro.25.112701.142758) 12052905

[63] Herring BE , Nicoll RA . 2016 Long-term potentiation: from CaMKII to AMPA receptor trafficking. Annu. Rev. Physiol. **78** , 351–365. (10.1146/annurev-physiol-021014-071753) 26863325

[64] Hayashi Y , Shi SH , Esteban JA , Piccini A , Poncer JC , Malinow R . 2000 Driving AMPA receptors into synapses by LTP and CaMKII: requirement for GluR1 and PDZ domain interaction. Science **287** , 2262–2267. (10.1126/science.287.5461.2262) 10731148

[65] Matsuzaki M , Honkura N , Ellis-Davies GCR , Kasai H . 2004 Structural basis of long-term potentiation in single dendritic spines. Nature **429** , 761–766. (10.1038/nature02617) 15190253 PMC4158816

[66] Shi S , Hayashi Y , Esteban JA , Malinow R . 2001 Subunit-specific rules governing AMPA receptor trafficking to synapses in hippocampal pyramidal neurons. Cell **105** , 331–343. (10.1016/s0092-8674(01)00321-x) 11348590

[67] Gerges NZ , Backos DS , Rupasinghe CN , Spaller MR , Esteban JA . 2006 Dual role of the exocyst in AMPA receptor targeting and insertion into the postsynaptic membrane. EMBO J. **25** , 1623–1634. (10.1038/sj.emboj.7601065) 16601687 PMC1440842

[68] Kennedy MJ , Davison IG , Robinson CG , Ehlers MD . 2010 Syntaxin-4 defines a domain for activity-dependent exocytosis in dendritic spines. Cell **141** , 524–535. (10.1016/j.cell.2010.02.042) 20434989 PMC2874581

[69] Penn AC , Zhang CL , Georges F , Royer L , Breillat C , Hosy E , Petersen JD , Humeau Y , Choquet D . 2017 Hippocampal LTP and contextual learning require surface diffusion of AMPA receptors. Nature **549** , 384–388. (10.1038/nature23658) 28902836 PMC5683353

[70] Getz AM *et al* . 2022 High-resolution imaging and manipulation of endogenous AMPA receptor surface mobility during synaptic plasticity and learning. Sci. Adv. **8** , eabm5298. (10.1126/sciadv.abm5298) 35895810 PMC9328687

[71] Lu J , Helton TD , Blanpied TA , Rácz B , Newpher TM , Weinberg RJ , Ehlers MD . 2007 Postsynaptic positioning of endocytic zones and AMPA receptor cycling by physical coupling of dynamin-3 to homer. Neuron **55** , 874–889. (10.1016/j.neuron.2007.06.041) 17880892 PMC2597538

[72] Rácz B , Blanpied TA , Ehlers MD , Weinberg RJ . 2004 Lateral organization of endocytic machinery in dendritic spines. Nat. Neurosci. **7** , 917–918. (10.1038/nn1303) 15322548

[73] Hiester BG , Becker MI , Bowen AB , Schwartz SL , Kennedy MJ . 2018 Mechanisms and role of dendritic membrane trafficking for long-term potentiation. Front. Cell. Neurosci. **12** , 391. (10.3389/fncel.2018.00391) 30425622 PMC6218485

[74] Hansen KB *et al* . 2021 Structure, function, and pharmacology of glutamate receptor ion channels. Pharmacol. Rev. **73** , 298–487. (10.1124/pharmrev.120.000131) 34753794 PMC8626789

[75] Plant K , Pelkey KA , Bortolotto ZA , Morita D , Terashima A , McBain CJ , Collingridge GL , Isaac JTR . 2006 Transient incorporation of native GluR2-lacking AMPA receptors during hippocampal long-term potentiation. Nat. Neurosci. **9** , 602–604. (10.1038/nn1678) 16582904

[76] Guire ES , Oh MC , Soderling TR , Derkach VA . 2008 Recruitment of calcium-permeable AMPA receptors during synaptic potentiation is regulated by CaM-kinase I. J. Neurosci. **28** , 6000–6009. (10.1523/JNEUROSCI.0384-08.2008) 18524905 PMC2671029

[77] Park P , Sanderson TM , Amici M , Choi SL , Bortolotto ZA , Zhuo M , Kaang BK , Collingridge GL . 2016 Calcium-permeable AMPA receptors mediate the induction of the protein kinase A-Dependent Component of Long-Term Potentiation in the Hippocampus. J. Neurosci. **36** , 622–631. (10.1523/JNEUROSCI.3625-15.2016) 26758849 PMC4710778

[78] Purkey AM , Dell’Acqua ML . 2020 Phosphorylation-dependent regulation of Ca^2+^-permeable AMPA receptors during hippocampal synaptic plasticity. Front. Synaptic Neurosci. **12** , 8. (10.3389/fnsyn.2020.00008) 32292336 PMC7119613

[79] Adesnik H , Nicoll RA . 2007 Conservation of glutamate receptor 2-containing AMPA receptors during long-term potentiation. J. Neurosci. **27** , 4598–4602. (10.1523/JNEUROSCI.0325-07.2007) 17460072 PMC6672988

[80] Gray EE , Fink AE , Sariñana J , Vissel B , O’Dell TJ . 2007 Long-term potentiation in the hippocampal CA1 region does not require insertion and activation of GluR2-lacking AMPA receptors. J. Neurophysiol. **98** , 2488–2492. (10.1152/jn.00473.2007) 17652419

[81] Granger AJ , Shi Y , Lu W , Cerpas M , Nicoll RA . 2013 LTP requires a reserve pool of glutamate receptors independent of subunit type. Nature **493** , 495–500. (10.1038/nature11775) 23235828 PMC3998843

[82] Savtchenko LP , Rusakov DA . 2014 Moderate AMPA receptor clustering on the nanoscale can efficiently potentiate synaptic current. Phil. Trans. R. Soc. B **369** , 20130167. (10.1098/rstb.2013.0167) 24298165 PMC3843895

[83] Sinnen BL , Bowen AB , Forte JS , Hiester BG , Crosby KC , Gibson ES , Dell’Acqua ML , Kennedy MJ . 2017 Optogenetic control of synaptic composition and function. Neuron **93** , 646–660. (10.1016/j.neuron.2016.12.037) 28132827 PMC5300939

[84] Liu G , Choi S , Tsien RW . 1999 Variability of neurotransmitter concentration and nonsaturation of postsynaptic AMPA receptors at synapses in hippocampal cultures and slices. Neuron **22** , 395–409. (10.1016/s0896-6273(00)81099-5) 10069344

[85] Franks KM , Stevens CF , Sejnowski TJ . 2003 Independent sources of quantal variability at single glutamatergic synapses. J. Neurosci. **23** , 3186–3195. (10.1523/JNEUROSCI.23-08-03186.2003) 12716926 PMC2944019

[86] Tarusawa E , Matsui K , Budisantoso T , Molnár E , Watanabe M , Matsui M , Fukazawa Y , Shigemoto R . 2009 Input-specific intrasynaptic arrangements of ionotropic glutamate receptors and their impact on postsynaptic responses. J. Neurosci. **29** , 12896–12908. (10.1523/JNEUROSCI.6160-08.2009) 19828804 PMC6665298

[87] Compans B , Choquet D , Hosy E . 2016 Review on the role of AMPA receptor nano-organization and dynamic in the properties of synaptic transmission. Neurophotonics **3** , 041811. (10.1117/1.NPh.3.4.041811) 27981061 PMC5109202

[88] MacGillavry HD , Song Y , Raghavachari S , Blanpied TA . 2013 Nanoscale scaffolding domains within the postsynaptic density concentrate synaptic AMPA receptors. Neuron **78** , 615–622. (10.1016/j.neuron.2013.03.009) 23719161 PMC3668352

[89] Nair D , Hosy E , Petersen JD , Constals A , Giannone G , Choquet D , Sibarita JB . 2013 Super-resolution imaging reveals that AMPA receptors inside synapses are dynamically organized in nanodomains regulated by PSD95. J. Neurosci. **33** , 13204–13224. (10.1523/JNEUROSCI.2381-12.2013) 23926273 PMC6619720

[90] Broadhead MJ *et al* . 2016 PSD95 nanoclusters are postsynaptic building blocks in hippocampus circuits. Sci. Rep. **6** , 24626. (10.1038/srep24626) 27109929 PMC4842999

[91] Fukata Y , Dimitrov A , Boncompain G , Vielemeyer O , Perez F , Fukata M . 2013 Local palmitoylation cycles define activity-regulated postsynaptic subdomains. J. Cell Biol. **202** , 145–161. (10.1083/jcb.201302071) 23836932 PMC3704990

[92] Goncalves J *et al* . 2020 Nanoscale co-organization and coactivation of AMPAR, NMDAR, and mGluR at excitatory synapses. Proc. Natl Acad. Sci. USA **117** , 14503–14511. (10.1073/pnas.1922563117) 32513712 PMC7321977

[93] Haas KT *et al* . 2018 Pre-post synaptic alignment through neuroligin-1 tunes synaptic transmission efficiency. Elife **7** , e31755. (10.7554/eLife.31755) 30044218 PMC6070337

[94] Chen H , Tang AH , Blanpied TA . 2018 Subsynaptic spatial organization as a regulator of synaptic strength and plasticity. Curr. Opin. Neurobiol. **51** , 147–153, (10.1016/j.conb.2018.05.004) 29902592 PMC6295321

[95] Li S *et al* . 2021 Asynchronous release sites align with NMDA receptors in mouse hippocampal synapses. Nat. Commun. **12** , 677. (10.1038/s41467-021-21004-x) 33514725 PMC7846561

[96] Hosokawa T *et al* . 2021 CaMKII activation persistently segregates postsynaptic proteins via liquid phase separation. Nat. Neurosci. **24** , 777–785. (10.1038/s41593-021-00843-3) 33927400

[97] Choquet D , Hosy E . 2020 AMPA receptor nanoscale dynamic organization and synaptic plasticities. Curr. Opin. Neurobiol. **63** , 137–145. (10.1016/j.conb.2020.04.003) 32416471

[98] Raghavachari S , Lisman JE . 2004 Properties of quantal transmission at CA1 synapses. J. Neurophysiol. **92** , 2456–2467. (10.1152/jn.00258.2004) 15115789

[99] Tang AH , Chen H , Li TP , Metzbower SR , MacGillavry HD , Blanpied TA . 2016 A trans-synaptic nanocolumn aligns neurotransmitter release to receptors. Nature **536** , 210–214. (10.1038/nature19058) 27462810 PMC5002394

[100] Hruska M , Cain RE , Dalva MB . 2022 Nanoscale rules governing the organization of glutamate receptors in spine synapses are subunit specific. Nat. Commun. **13** , 920. (10.1038/s41467-022-28504-4) 35177616 PMC8854560

[101] Scheefhals N , Westra M , MacGillavry HD . 2023 mGluR5 is transiently confined in perisynaptic nanodomains to shape synaptic function. Nat. Commun. **14** , 244. (10.1038/s41467-022-35680-w) 36646691 PMC9842668

[102] Matsui A , *et al* . 2024 Cryo-electron Tomographic investigation of native hippocampal glutamatergic synapses. BioRxiv. (10.1101/2024.04.01.587595) PMC11534335 39495821

[103] Martinez-Sanchez A , Laugks U , Kochovski Z , Papantoniou C , Zinzula L , Baumeister W , Lučić V . 2021 Trans-synaptic assemblies link synaptic vesicles and neuroreceptors. Sci. Adv. **7** , eabe6204. (10.1126/sciadv.abe6204) 33674312 PMC7935360

[104] Perez de Arce K *et al* . 2015 Topographic mapping of the synaptic cleft into adhesive nanodomains. Neuron **88** , 1165–1172. (10.1016/j.neuron.2015.11.011) 26687224 PMC4687029

[105] Lucić V , Yang T , Schweikert G , Förster F , Baumeister W . 2005 Morphological characterization of molecular complexes present in the synaptic cleft. Structure **13** , 423–434. (10.1016/j.str.2005.02.005) 15766544

[106] Jung JH , Chen X , Reese TS . 2023 Cryo-EM tomography and automatic segmentation delineate modular structures in the postsynaptic density. Front. Synaptic Neurosci. **15** , 1123564. (10.3389/fnsyn.2023.1123564) 37091879 PMC10117989

[107] Cole AA , Reese TS . 2023 Transsynaptic assemblies link domains of presynaptic and postsynaptic intracellular structures across the synaptic cleft. J. Neurosci. **43** , 5883–5892. (10.1523/JNEUROSCI.2195-22.2023) 37369583 PMC10436760

[108] Sun R , Allen JP , Wilson L , Haider M , Alten B , Zhou Z , Wang X , Zhou Q . 2024 Cryo-electron tomography reveals postsynaptic Nanoblocks in excitatory synapses. bioRxiv. (10.1101/2023.05.12.540562)

[109] Biederer T , Kaeser PS , Blanpied TA . 2017 Transcellular nanoalignment of synaptic function. Neuron **96** , 680–696. (10.1016/j.neuron.2017.10.006) 29096080 PMC5777221

[110] Südhof TC . 2008 Neuroligins and neurexins link synaptic function to cognitive disease. Nature **455** , 903–911. (10.1038/nature07456) 18923512 PMC2673233

[111] Lloyd BA , Han Y , Roth R , Zhang B , Aoto J . 2023 Neurexin-3 subsynaptic densities are spatially distinct from Neurexin-1 and essential for excitatory synapse nanoscale organization in the hippocampus. Nat. Commun. **14** , 4706. (10.1038/s41467-023-40419-2) 37543682 PMC10404257

[112] Chamma I , Levet F , Sibarita JB , Sainlos M , Thoumine O . 2016 Nanoscale organization of synaptic adhesion proteins revealed by single-molecule localization microscopy. Neurophotonics **3** , 041810. (10.1117/1.NPh.3.4.041810) 27872870 PMC5093229

[113] Ramsey AM , Tang AH , LeGates TA , Gou XZ , Carbone BE , Thompson SM , Biederer T , Blanpied TA . 2021 Subsynaptic positioning of AMPARs by LRRTM2 controls synaptic strength. Sci. Adv. **7** , eabf3126. (10.1126/sciadv.abf3126) 34417170 PMC8378824

[114] Schoch S , Castillo PE , Jo T , Mukherjee K , Geppert M , Wang Y , Schmitz F , Malenka RC , Südhof TC . 2002 RIM1α forms a protein scaffold for regulating neurotransmitter release at the active zone. Nature **415** , 321–326. (10.1038/415321a) 11797009

[115] Serra-Pagès C , Medley QG , Tang M , Hart A , Streuli M . 1998 Liprins, a family of LAR transmembrane protein-tyrosine phosphatase-interacting proteins. J. Biol. Chem. **273** , 15611–15620. (10.1074/jbc.273.25.15611) 9624153

[116] Beaubien F , Raja R , Kennedy TE , Fournier AE , Cloutier JF . 2016 Slitrk1 is localized to excitatory synapses and promotes their development. Sci. Rep. **6** , 27343. (10.1038/srep27343) 27273464 PMC4895136

[117] Choi Y *et al* . 2016 SALM5 trans-synaptically interacts with LAR-RPTPs in a splicing-dependent manner to regulate synapse development. Sci. Rep. **6** , 26676. (10.1038/srep26676) 27225731 PMC4881023

[118] Li Y *et al* . 2015 Splicing-dependent trans-synaptic SALM3-LAR-RPTP interactions regulate excitatory synapse development and locomotion. Cell Rep. **12** , 1618–1630. (10.1016/j.celrep.2015.08.002) 26321637 PMC4578660

[119] Woo J , Kwon SK , Choi S , Kim S , Lee JR , Dunah AW , Sheng M , Kim E . 2009 Trans-synaptic adhesion between NGL-3 and LAR regulates the formation of excitatory synapses. Nat. Neurosci. **12** , 428–437. (10.1038/nn.2279) 19252495

[120] Yim YS , Kwon Y , Nam J , Yoon HI , Lee K , Kim DG , Kim E , Kim CH , Ko J . 2013 Slitrks control excitatory and inhibitory synapse formation with LAR receptor protein tyrosine phosphatases. Proc. Natl Acad. Sci. USA **110** , 4057–4062. (10.1073/pnas.1209881110) 23345436 PMC3593915

[121] Takeichi M . 2007 The cadherin superfamily in neuronal connections and interactions. Nat. Rev. Neurosci. **8** , 11–20. (10.1038/nrn2043) 17133224

[122] Brasch J , Harrison OJ , Honig B , Shapiro L . 2012 Thinking outside the cell: how cadherins drive adhesion. Trends Cell Biol. **22** , 299–310. (10.1016/j.tcb.2012.03.004) 22555008 PMC3385655

[123] Friedman LG , Benson DL , Huntley GW . 2015 Cadherin-based transsynaptic networks in establishing and modifying neural connectivity. Curr. Top. Dev. Biol. **112** , 415–465. (10.1016/bs.ctdb.2014.11.025) 25733148 PMC4418560

[124] Saglietti L *et al* . 2007 Extracellular interactions between GluR2 and N-Cadherin in spine regulation. Neuron **54** , 461–477. (10.1016/j.neuron.2007.04.012) 17481398

[125] Pozo K , Cingolani LA , Bassani S , Laurent F , Passafaro M , Goda Y . 2012 β3 integrin interacts directly with GluA2 AMPA receptor subunit and regulates AMPA receptor expression in hippocampal neurons. Proc. Natl Acad. Sci. USA **109** , 1323–1328. (10.1073/pnas.1113736109) 22232691 PMC3268285

[126] Hynes RO . 2002 Integrins: bidirectional, allosteric signaling machines. Cell **110** , 673–687. (10.1016/s0092-8674(02)00971-6) 12297042

[127] Reti IM *et al* . 2008 Activity-dependent secretion of neuronal activity regulated pentraxin from vasopressin neurons into the systemic circulation. Neuroscience **151** , 352–360. (10.1016/j.neuroscience.2007.10.040) 18082971 PMC2342909

[128] Lee SJ , Wei M , Zhang C , Maxeiner S , Pak C , Calado Botelho S , Trotter J , Sterky FH , Südhof TC . 2017 Presynaptic neuronal pentraxin receptor organizes excitatory and inhibitory synapses. J. Neurosci. **37** , 1062–1080. (10.1523/JNEUROSCI.2768-16.2016) 27986928 PMC5296791

[129] Sia GM , Béïque JC , Rumbaugh G , Cho R , Worley PF , Huganir RL . 2007 Interaction of the N-terminal domain of the AMPA receptor GluR4 subunit with the neuronal pentraxin NP1 mediates GluR4 synaptic recruitment. Neuron **55** , 87–102. (10.1016/j.neuron.2007.06.020) 17610819

[130] Hoppa MB , Lana B , Margas W , Dolphin AC , Ryan TA . 2012 α2δ expression sets presynaptic calcium channel abundance and release probability. Nature **486** , 122–125. (10.1038/nature11033) 22678293 PMC3376018

[131] Schneider R , Hosy E , Kohl J , Klueva J , Choquet D , Thomas U , Voigt A , Heine M . 2015 Mobility of calcium channels in the presynaptic membrane. Neuron **86** , 672–679. (10.1016/j.neuron.2015.03.050) 25892305

[132] Boudkkazi S *et al* . 2023 A Noelin-organized extracellular network of proteins required for constitutive and context-dependent anchoring of AMPA-receptors. Neuron **111** , 2544–2556. (10.1016/j.neuron.2023.07.013) 37591201 PMC10441612

[133] Pandya NJ *et al* . 2018 Noelin1 affects lateral mobility of synaptic AMPA receptors. Cell Rep. **24** , 1218–1230, (10.1016/j.celrep.2018.06.102) 30067977 PMC6088136

[134] Hruska M , Henderson N , Le Marchand SJ , Jafri H , Dalva MB . 2018 Synaptic nanomodules underlie the organization and plasticity of spine synapses. Nat. Neurosci. **21** , 671–682. (10.1038/s41593-018-0138-9) 29686261 PMC5920789

[135] Choquet D . 2018 Linking nanoscale dynamics of AMPA receptor organization to plasticity of excitatory synapses and learning. J. Neurosci. **38** , 9318–9329. (10.1523/JNEUROSCI.2119-18.2018) 30381423 PMC6705996

[136] Chen H , Roth RH , Lopez-Ortega E , Tan HL , Huganir RL . 2021 AMPA receptors exist in tunable mobile and immobile synaptic fractions in vivo. eNeuro **8** . (10.1523/ENEURO.0015-21.2021) PMC8143022 33906969

[137] Frischknecht R , Heine M , Perrais D , Seidenbecher CI , Choquet D , Gundelfinger ED . 2009 The brain extracellular matrix limits lateral diffusion of AMPA receptors and modulates short-term synaptic plasticity. Nat. Neurosci **12** , 897–904. (10.1038/nn.2338) 19483686

[138] Choquet D . 2010 Fast AMPAR trafficking for a high-frequency synaptic transmission. Eur. J. Neurosci. **32** , 250–260. (10.1111/j.1460-9568.2010.07350.x) 20646044

[139] Tardin C , Cognet L , Bats C , Lounis B , Choquet D . 2003 Direct imaging of lateral movements of AMPA receptors inside synapses. EMBO J. **22** , 4656–4665. (10.1093/emboj/cdg463) 12970178 PMC212729

[140] Li TP , Song Y , MacGillavry HD , Blanpied TA , Raghavachari S . 2016 Protein crowding within the postsynaptic density can impede the escape of membrane proteins. J. Neurosci. **36** , 4276–4295. (10.1523/JNEUROSCI.3154-15.2016) 27076425 PMC4829651

[141] Renner M , Schweizer C , Bannai H , Triller A , Lévi S . 2012 Diffusion barriers constrain receptors at synapses. PLoS One **7** , e43032. (10.1371/journal.pone.0043032) 22912780 PMC3418229

[142] Groc L , Choquet D . 2006 AMPA and NMDA glutamate receptor trafficking: multiple roads for reaching and leaving the synapse. Cell Tissue Res. **326** , 423–438. (10.1007/s00441-006-0254-9) 16847641

[143] Compans B *et al* . 2021 NMDAR-dependent long-term depression is associated with increased short term plasticity through autophagy mediated loss of PSD-95. Nat. Commun. **12** , 2849. (10.1038/s41467-021-23133-9) 33990590 PMC8121912

[144] Groc L , Choquet D , Chaouloff F . 2008 The stress hormone corticosterone conditions AMPAR surface trafficking and synaptic potentiation. Nat. Neurosci. **11** , 868–870. (10.1038/nn.2150) 18622402

[145] Bayer KU , De Koninck P , Leonard AS , Hell JW , Schulman H . 2001 Interaction with the NMDA receptor locks CaMKII in an active conformation. Nature **411** , 801–805. (10.1038/35081080) 11459059

[146] Groc L , Lafourcade M , Heine M , Renner M , Racine V , Sibarita JB , Lounis B , Choquet D , Cognet L . 2007 Surface trafficking of neurotransmitter receptor: comparison between single-molecule/quantum dot strategies. J. Neurosci. **27** , 12433–12437. (10.1523/JNEUROSCI.3349-07.2007) 18003820 PMC6673310

[147] Shepherd JD , Huganir RL . 2007 The cell biology of synaptic plasticity: AMPA receptor trafficking. Annu. Rev. Cell Dev. Biol. **23** , 613–643. (10.1146/annurev.cellbio.23.090506.123516) 17506699

[148] Barry MF , Ziff EB . 2002 Receptor trafficking and the plasticity of excitatory synapses. Curr. Opin. Neurobiol. **12** , 279–286. (10.1016/s0959-4388(02)00329-x) 12049934

[149] Diering GH , Huganir RL . 2018 The AMPA receptor code of synaptic plasticity. Neuron **100** , 314–329. (10.1016/j.neuron.2018.10.018) 30359599 PMC6214363

[150] Zhou Z *et al* . 2018 The C-terminal tails of endogenous GluA1 and GluA2 differentially contribute to hippocampal synaptic plasticity and learning. Nat. Neurosci. **21** , 50–62. (10.1038/s41593-017-0030-z) 29230056

[151] Watson JF , Pinggera A , Ho H , Greger IH . 2021 AMPA receptor anchoring at CA1 synapses is determined by N-terminal domain and TARP γ8 interactions. Nat. Commun. **12** , 5083. (10.1038/s41467-021-25281-4) 34426577 PMC8382838

[152] Díaz-Alonso J , Morishita W , Incontro S , Simms J , Holtzman J , Gill M , Mucke L , Malenka RC , Nicoll RA . 2020 Long-term potentiation is independent of the C-tail of the GluA1 AMPA receptor subunit. Elife **9** . (10.7554/eLife.58042) PMC7500950 32831170

[153] Liu A , Ji H , Ren Q , Meng Y , Zhang H , Collingride G , Xie W , Jia Z . 2020 The requirement of the C-terminal domain of GluA1 in different forms of long-term potentiation in the hippocampus is age-dependent. Front. Synaptic Neurosci. **12** , 588785. (10.3389/fnsyn.2020.588785) 33192442 PMC7661473

[154] Díaz-Alonso J , Nicoll RA . 2021 AMPA receptor trafficking and LTP: Carboxy-termini, amino-termini and TARPs. Neuropharmacology **197** , 108710, (10.1016/j.neuropharm.2021.108710) 34271016 PMC9122021

[155] Hangen E , Cordelières FP , Petersen JD , Choquet D , Coussen F . 2018 Neuronal activity and intracellular calcium levels regulate intracellular transport of newly synthesized AMPAR. Cell Rep. **24** , 1001–1012.(10.1016/j.celrep.2018.06.095) 30044968 PMC6083039

[156] Chen L , Chetkovich DM , Petralia RS , Sweeney NT , Kawasaki Y , Wenthold RJ , Bredt DS , Nicoll RA . 2000 Stargazin regulates synaptic targeting of AMPA receptors by two distinct mechanisms. Nature **408** , 936–943. (10.1038/35050030) 11140673

[157] Fukaya M , Yamazaki M , Sakimura K , Watanabe M . 2005 Spatial diversity in gene expression for VDCCγ subunit family in developing and adult mouse brains. Neurosci. Res. **53** , 376–383. (10.1016/j.neures.2005.08.009) 16171881

[158] Tomita S , Chen L , Kawasaki Y , Petralia RS , Wenthold RJ , Nicoll RA , Bredt DS . 2003 Functional studies and distribution define a family of transmembrane AMPA receptor regulatory proteins. J. Cell Biol. **161** , 805–816. (10.1083/jcb.200212116) 12771129 PMC2199354

[159] Constals A *et al* . 2015 Glutamate-induced AMPA receptor desensitization increases their mobility and modulates short-term plasticity through unbinding from stargazin. Neuron **85** , 787–803, (10.1016/j.neuron.2015.01.012) 25661182

[160] Hafner AS *et al* . 2015 Lengthening of the stargazin cytoplasmic tail increases synaptic transmission by promoting interaction to deeper domains of PSD-95. Neuron **86** , 475–489, (10.1016/j.neuron.2015.03.013) 25843401

[161] Tomita S , Fukata M , Nicoll RA , Bredt DS . 2004 Dynamic interaction of stargazin-like TARPs with cycling AMPA receptors at synapses. Science **303** , 1508–1511. (10.1126/science.1090262) 15001777

[162] Matsuda S , Kakegawa W , Budisantoso T , Nomura T , Kohda K , Yuzaki M . 2013 Stargazin regulates AMPA receptor trafficking through adaptor protein complexes during long-term depression. Nat. Commun. **4** , 2759. (10.1038/ncomms3759) 24217640

[163] El-Husseini AED *et al* . 2002 Synaptic strength regulated by palmitate cycling on PSD-95. Cell **108** , 849–863. (10.1016/s0092-8674(02)00683-9) 11955437

[164] Schnell E , Sizemore M , Karimzadegan S , Chen L , Bredt DS , Nicoll RA . 2002 Direct interactions between PSD-95 and stargazin control synaptic AMPA receptor number. Proc. Natl Acad. Sci. USA **99** , 13902–13907. (10.1073/pnas.172511199) 12359873 PMC129795

[165] Stein ELA , Chetkovich DM . 2010 Regulation of stargazin synaptic trafficking by C-terminal PDZ ligand phosphorylation in bidirectional synaptic plasticity. J. Neurochem. **113** , 42–53. (10.1111/j.1471-4159.2009.06529.x) 19968761 PMC4035483

[166] Nomura T , Kakegawa W , Matsuda S , Kohda K , Nishiyama J , Takahashi T , Yuzaki M . 2012 Cerebellar long-term depression requires dephosphorylation of TARP in Purkinje cells. Eur. J. Neurosci. **35** , 402–410. (10.1111/j.1460-9568.2011.07963.x) 22239345

[167] Louros SR , Hooks BM , Litvina L , Carvalho AL , Chen C . 2014 A role for stargazin in experience-dependent plasticity. Cell Reports **7** , 1614–1625. (10.1016/j.celrep.2014.04.054) 24882000 PMC4115130

[168] Louros SR , Caldeira GL , Carvalho AL . 2018 Stargazin dephosphorylation mediates homeostatic synaptic downscaling of excitatory synapses. Front. Mol. Neurosci. **11** , 328. (10.3389/fnmol.2018.00328) 30271322 PMC6146028

[169] Dakoji S , Tomita S , Karimzadegan S , Nicoll RA , Bredt DS . 2003 Interaction of transmembrane AMPA receptor regulatory proteins with multiple membrane associated guanylate kinases. Neuropharmacology **45** , 849–856. (10.1016/s0028-3908(03)00267-3) 14529722

[170] Choi J , Ko J , Park E , Lee JR , Yoon J , Lim S , Kim E . 2002 Phosphorylation of stargazin by protein kinase A regulates its interaction with PSD-95. J. Biol. Chem. **277** , 12359–12363. (10.1074/jbc.M200528200) 11805122

[171] Chetkovich DM , Chen L , Stocker TJ , Nicoll RA , Bredt DS . 2002 Phosphorylation of the postsynaptic density-95 (PSD-95)/Discs Large/Zona Occludens-1 Binding Site of Stargazin Regulates Binding to PSD-95 and Synaptic Targeting of AMPA Receptors. J. Neurosci. **22** , 5791–5796. (10.1523/JNEUROSCI.22-14-05791.2002) 12122038 PMC6757934

[172] Sainlos M , Tigaret C , Poujol C , Olivier NB , Bard L , Breillat C , Thiolon K , Choquet D , Imperiali B . 2011 Biomimetic divalent ligands for the acute disruption of synaptic AMPAR stabilization. Nat. Chem. Biol. **7** , 81–91. (10.1038/nchembio.498) 21186349

[173] Zeng M , Díaz-Alonso J , Ye F , Chen X , Xu J , Ji Z , Nicoll RA , Zhang M . 2019 Phase separation-mediated TARP/MAGUK complex condensation and AMPA receptor synaptic transmission. Neuron **104** , 529–543.(10.1016/j.neuron.2019.08.001) 31492534 PMC6842113

[174] Sumioka A , Yan D , Tomita S . 2010 TARP phosphorylation regulates synaptic AMPA receptors through lipid bilayers. Neuron **66** , 755–767. (10.1016/j.neuron.2010.04.035) 20547132 PMC2887694

[175] Zeng M , Chen X , Guan D , Xu J , Wu H , Tong P , Zhang M . 2018 Reconstituted postsynaptic density as a molecular platform for understanding synapse formation and plasticity. Cell **174** , 1172–1187.(10.1016/j.cell.2018.06.047) 30078712

[176] Zeng M , Shang Y , Araki Y , Guo T , Huganir RL , Zhang M . 2016 Phase transition in postsynaptic densities underlies formation of synaptic complexes and synaptic plasticity. Cell **166** , 1163–1175.(10.1016/j.cell.2016.07.008) 27565345 PMC5564291

[177] Xu W , Schlüter OM , Steiner P , Czervionke BL , Sabatini B , Malenka RC . 2008 Molecular dissociation of the role of PSD-95 in regulating synaptic strength and LTD. Neuron **57** , 248–262. (10.1016/j.neuron.2007.11.027) 18215622 PMC3147180

[178] Tomita S , Adesnik H , Sekiguchi M , Zhang W , Wada K , Howe JR , Nicoll RA , Bredt DS . 2005 Stargazin modulates AMPA receptor gating and trafficking by distinct domains. Nature **435** , 1052–1058. (10.1038/nature03624) 15858532

[179] Tsui J , Malenka RC . 2006 Substrate localization creates specificity in calcium/calmodulin-dependent protein kinase II signaling at synapses. J. Biol. Chem. **281** , 13794–13804. (10.1074/jbc.M600966200) 16551613

[180] Tomita S , Stein V , Stocker TJ , Nicoll RA , Bredt DS . 2005 Bidirectional synaptic plasticity regulated by phosphorylation of stargazin-like TARPs. Neuron **45** , 269–277. (10.1016/j.neuron.2005.01.009) 15664178

[181] Ribeiro LF , Catarino T , Santos SD , Benoist M , van Leeuwen JF , Esteban JA , Carvalho AL . 2014 Ghrelin triggers the synaptic incorporation of AMPA receptors in the hippocampus. Proc. Natl Acad. Sci. USA **111** , E149–58. (10.1073/pnas.1313798111) 24367106 PMC3890894

[182] Turrigiano GG . 2008 The self-tuning neuron: synaptic scaling of excitatory synapses. Cell **135** , 422–435. (10.1016/j.cell.2008.10.008) 18984155 PMC2834419

[183] Morimoto-Tomita M , Zhang W , Straub C , Cho CH , Kim KS , Howe JR , Tomita S . 2009 Autoinactivation of neuronal AMPA receptors via glutamate-regulated TARP interaction. Neuron **61** , 101–112. (10.1016/j.neuron.2008.11.009) 19146816 PMC2649795

[184] Nakagawa T , Cheng Y , Ramm E , Sheng M , Walz T . 2005 Structure and different conformational states of native AMPA receptor complexes. Nature **433** , 545–549. (10.1038/nature03328) 15690046

[185] Semenov A , Möykkynen T , Coleman SK , Korpi ER , Keinänen K . 2012 Autoinactivation of the stargazin–AMPA receptor complex: subunit-dependency and independence from physical dissociation. PLoS One **7** , e49282. (10.1371/journal.pone.0049282) 23166629 PMC3498123

[186] Shaikh SA , Dolino DM , Lee G , Chatterjee S , MacLean DM , Flatebo C , Landes CF , Jayaraman V . 2016 Stargazin modulation of AMPA receptors. Cell Reports **17** , 328–335. (10.1016/j.celrep.2016.09.014) 27705782 PMC5088782

[187] Coombs ID , MacLean DM , Jayaraman V , Farrant M , Cull-Candy SG . 2017 Dual effects of TARP γ-2 on glutamate efficacy can account for AMPA receptor autoinactivation. Cell Rep. **20** , 1123–1135, (10.1016/j.celrep.2017.07.014) 28768197 PMC5554777

[188] Baranovic J , Plested AJ . 2018 Auxiliary subunits keep AMPA receptors compact during activation and desensitization. Elife **7** , e40548. (10.7554/eLife.40548) 30520730 PMC6324883

[189] Zhang D , Ivica J , Krieger JM , Ho H , Yamashita K , Stockwell I , Baradaran R , Cais O , Greger IH . 2023 Structural mobility tunes signalling of the GluA1 AMPA glutamate receptor. Nature **621** , 877–882. (10.1038/s41586-023-06528-0) 37704721 PMC10533411

[190] Herguedas B *et al* . 2022 Mechanisms underlying TARP modulation of the GluA1/2-γ8 AMPA receptor. Nat. Commun. **13** , 734. (10.1038/s41467-022-28404-7) 35136046 PMC8826358

[191] Twomey EC , Yelshanskaya MV , Grassucci RA , Frank J , Sobolevsky AI . 2017 Structural bases of desensitization in AMPA receptor-auxiliary subunit complexes. Neuron **94** , 569–580.(10.1016/j.neuron.2017.04.025) 28472657 PMC5492975

[192] Chen S , Zhao Y , Wang Y , Shekhar M , Tajkhorshid E , Gouaux E . 2017 Activation and desensitization mechanism of AMPA receptor-TARP complex by Cryo-EM. Cell **170** , 1234–1246.(10.1016/j.cell.2017.07.045) 28823560 PMC5621841

[193] Fukaya M *et al* *.* 2006 Abundant distribution of TARP γ-8 in synaptic and extrasynaptic surface of hippocampal neurons and its major role in AMPA receptor expression on spines and dendrites. Eur. J. Neurosci. **24** , 2177–2190. (10.1111/j.1460-9568.2006.05081.x) 17074043

[194] Rouach N *et al* . 2005 TARP γ-8 controls hippocampal AMPA receptor number, distribution and synaptic plasticity. Nat. Neurosci. **8** , 1525–1533. (10.1038/nn1551) 16222232

[195] Khodosevich K , Jacobi E , Farrow P , Schulmann A , Rusu A , Zhang L , Sprengel R , Monyer H , von Engelhardt J . 2014 Coexpressed auxiliary subunits exhibit distinct modulatory profiles on AMPA receptor function. Neuron **83** , 601–615. (10.1016/j.neuron.2014.07.004) 25066086

[196] Inamura M , Itakura M , Okamoto H , Hoka S , Mizoguchi A , Fukazawa Y , Shigemoto R , Yamamori S , Takahashi M . 2006 Differential localization and regulation of stargazin-like protein, γ-8 and stargazin in the plasma membrane of hippocampal and cortical neurons. Neurosci. Res. **55** , 45–53. (10.1016/j.neures.2006.01.004) 16516319

[197] Park J , Chávez AE , Mineur YS , Morimoto-Tomita M , Lutzu S , Kim KS , Picciotto MR , Castillo PE , Tomita S . 2016 CaMKII phosphorylation of TARPγ-8 is a mediator of LTP and learning and memory. Neuron **92** , 75–83, (10.1016/j.neuron.2016.09.002) 27667007 PMC5059846

[198] Itakura M , Watanabe I , Sugaya T , Takahashi M . 2014 Direct association of the unique C-terminal tail of transmembrane AMPA receptor regulatory protein γ-8 with calcineurin. FEBS J. **281** , 1366–1378. (10.1111/febs.12708) 24418105

[199] Sumioka A , Brown TE , Kato AS , Bredt DS , Kauer JA , Tomita S . 2011 PDZ binding of TARPγ-8 controls synaptic transmission but not synaptic plasticity. Nat. Neurosci. **14** , 1410–1412. (10.1038/nn.2952) 22002768 PMC3206644

[200] Sheng N , Bemben MA , Díaz-Alonso J , Tao W , Shi YS , Nicoll RA . 2018 LTP requires postsynaptic PDZ-domain interactions with glutamate receptor/auxiliary protein complexes. Proc. Natl Acad. Sci. USA **115** , 3948–3953. (10.1073/pnas.1800719115) 29581259 PMC5899490

[201] Bessa-Neto D *et al* . 2021 Bioorthogonal labeling of transmembrane proteins with non-canonical amino acids unveils masked epitopes in live neurons. Nat. Commun. **12** , 6715. (10.1038/s41467-021-27025-w) 34795271 PMC8602626

[202] Tardin C , Cognet L , Bats C , Lounis B , Choquet D . 2003 Direct imaging of lateral movements of AMPA receptors inside synapses. EMBO J. **22** , 4656–4665. (10.1093/emboj/cdg463) 12970178 PMC212729

[203] Martin S , Henley JM , Holman D , Zhou M , Wiegert O , van Spronsen M , Joëls M , Hoogenraad CC , Krugers HJ . 2009 Corticosterone alters AMPAR mobility and facilitates bidirectional synaptic plasticity. PLoS One **4** , e4714. (10.1371/journal.pone.0004714) 19305644 PMC2659165

[204] Choquet D , Ku G , Cassard S , Malissen B , Korn H , Fridman WH , Bonnerot C . 1994 Different patterns of calcium signaling triggered through two components of the B lymphocyte antigen receptor. J. Biol. Chem **269** , 6491–6497. (doi:10.1016/S0021-9258(17)37398-2) 7509805

[205] Haselmann H *et al* . 2018 Human autoantibodies against the AMPA receptor subunit GluA2 induce receptor reorganization and memory dysfunction. Neuron **100** , 91–105.(10.1016/j.neuron.2018.07.048) 30146304

[206] El Oussini H *et al* . 2023 CA3 hippocampal synaptic plasticity supports ripple physiology during memory consolidation. Nat. Commun. **14** , 8312. (10.1038/s41467-023-42969-x) 38097535 PMC10721822

[207] Campelo T , Augusto E , Chenouard N , de Miranda A , Kouskoff V , Camus C , Choquet D , Gambino F . 2020 AMPAR-dependent synaptic plasticity initiates cortical remapping and adaptive behaviors during sensory experience. Cell Rep. **32** , 108097. (10.1016/j.celrep.2020.108097) 32877679 PMC7487777

[208] Polenghi A , Nieus T , Guazzi S , Gorostiza P , Petrini EM , Barberis A . 2020 Kainate receptor activation shapes short-term synaptic plasticity by controlling receptor lateral mobility at glutamatergic synapses. Cell Reports **31** , 107735. (10.1016/j.celrep.2020.107735) 32521260 PMC7296349

[209] de Luca E , Ravasenga T , Petrini EM , Polenghi A , Nieus T , Guazzi S , Barberis A . 2017 Inter-synaptic lateral diffusion of GABAA receptors shapes inhibitory synaptic currents. Neuron **95** , 63–69.(10.1016/j.neuron.2017.06.022) 28683270 PMC5500312

[210] Dupuis JP *et al* . 2014 Surface dynamics of GluN2B-NMDA receptors controls plasticity of maturing glutamate synapses. EMBO J. **33** , 842–861. (10.1002/embj.201386356) 24591565 PMC4194110

[211] Murphy-Royal C , Dupuis JP , Varela JA , Panatier A , Pinson B , Baufreton J , Groc L , Oliet SHR . 2015 Surface diffusion of astrocytic glutamate transporters shapes synaptic transmission. Nat. Neurosci. **18** , 219–226. (10.1038/nn.3901) 25581361

[212] Ciappelloni S *et al* . 2019 Aquaporin-4 surface trafficking regulates astrocytic process motility and synaptic activity in health and autoimmune disease. Cell Rep. **27** , 3860–3872.(10.1016/j.celrep.2019.05.097) 31242419

[213] Crosby KC , Gookin SE , Garcia JD , Hahm KM , Dell’Acqua ML , Smith KR . 2019 Nanoscale subsynaptic domains underlie the organization of the inhibitory synapse. Cell Rep. **26** , 3284–3297.(10.1016/j.celrep.2019.02.070) 30893601 PMC6529211

[214] Kellermayer B *et al* . 2018 Differential nanoscale topography and functional role of GluN2-NMDA receptor subtypes at glutamatergic synapses. Neuron **100** , 106–119.(10.1016/j.neuron.2018.09.012) 30269991

[215] Suzuki K *et al* . 2020 A synthetic synaptic organizer protein restores glutamatergic neuronal circuits. Science **369** , eabb4853. (10.1126/science.abb4853) 32855309 PMC7116145

[216] Rimbault C *et al* . 2024 Engineering paralog-specific PSD-95 recombinant binders as minimally interfering multimodal probes for advanced imaging techniques. Elife **13** , e69620. (10.7554/eLife.69620) 38167295 PMC10803022

[217] Tullis JE *et al* . 2023 LTP induction by structural rather than enzymatic functions of CaMKII. Nature **621** , 146–153. (10.1038/s41586-023-06465-y) 37648853 PMC10482691

[218] Gamache TR , Araki Y , Huganir RL . 2020 Twenty years of SynGAP research: from synapses to cognition. J. Neurosci. **40** , 1596–1605. (10.1523/JNEUROSCI.0420-19.2020) 32075947 PMC7046327

[219] Araki Y *et al* . 2024 SynGAP regulates synaptic plasticity and cognition independently of its catalytic activity. Science **383** , eadk1291. (10.1126/science.adk1291) 38422154 PMC11188940

[220] Kim JH , Liao D , Lau LF , Huganir RL . 1998 SynGAP: a synaptic RasGAP that associates with the PSD-95/SAP90 protein family. Neuron **20** , 683–691. (10.1016/s0896-6273(00)81008-9) 9581761

[221] Chen HJ , Rojas-Soto M , Oguni A , Kennedy MB . 1998 A synaptic Ras-GTPase activating protein (p135 SynGAP) inhibited by CaM kinase II. Neuron **20** , 895–904. (10.1016/s0896-6273(00)80471-7) 9620694

[222] Araki Y , Zeng M , Zhang M , Huganir RL . 2015 Rapid dispersion of SynGAP from synaptic spines triggers AMPA receptor insertion and spine enlargement during LTP. Neuron **85** , 173–189, (10.1016/j.neuron.2014.12.023) 25569349 PMC4428669

[223] Watson JF , Ho H , Greger IH . 2017 Synaptic transmission and plasticity require AMPA receptor anchoring via its N-terminal domain. Elife **6** , e23024. (10.7554/eLife.23024) 28290985 PMC5370185

[224] Díaz-Alonso J , Sun YJ , Granger AJ , Levy JM , Blankenship SM , Nicoll RA . 2017 Subunit-specific role for the amino-terminal domain of AMPA receptors in synaptic targeting. Proc. Natl Acad. Sci. USA **114** , 7136–7141. (10.1073/pnas.1707472114) 28630296 PMC5502653

[225] Jiang CH , Wei M , Zhang C , Shi YS . 2021 The amino-terminal domain of GluA1 mediates LTP maintenance via interaction with neuroplastin-65. Proc. Natl Acad. Sci. USA **118** , e2019194118. (10.1073/pnas.2019194118) 33627404 PMC7936340

